# SpineTool is an open-source software for analysis of morphology of dendritic spines

**DOI:** 10.1038/s41598-023-37406-4

**Published:** 2023-06-29

**Authors:** Ekaterina Pchitskaya, Peter Vasiliev, Daria Smirnova, Vyacheslav Chukanov, Ilya Bezprozvanny

**Affiliations:** 1 Laboratory of Molecular Neurodegeneration, Peter the Great St. Petersburg Polytechnic University, Khlopina St. 11, St. Petersburg, Russia 194021; 2 Department of Applied Mathematics, Peter the Great St. Petersburg Polytechnic University, Polytechnicheskaya St. 29, St. Petersburg, Russia 195251; 3 Department of Physiology, UT Southwestern Medical Center at Dallas, Dallas, TX 75390 USA

**Keywords:** 3-D reconstruction, Fluorescence imaging, Optical imaging, Translational research, Neurological disorders

## Abstract

Dendritic spines form most excitatory synaptic inputs in neurons and these spines are altered in many neurodevelopmental and neurodegenerative disorders. Reliable methods to assess and quantify dendritic spines morphology are needed, but most existing methods are subjective and labor intensive. To solve this problem, we developed an open-source software that allows segmentation of dendritic spines from 3D images, extraction of their key morphological features, and their classification and clustering. Instead of commonly used spine descriptors based on numerical metrics we used chord length distribution histogram (CLDH) approach. CLDH method depends on distribution of lengths of chords randomly generated within dendritic spines volume. To achieve less biased analysis, we developed a classification procedure that uses machine-learning algorithm based on experts’ consensus and machine-guided clustering tool. These approaches to unbiased and automated measurements, classification and clustering of synaptic spines that we developed should provide a useful resource for a variety of neuroscience and neurodegenerative research applications.

## Introduction

Synapse is a place of contact between two neurons, serving to transmit information from cell to cell. Most synapses are formed between the axonal bouton and the dendritic spine, which is a specialized protrusion from the dendritic membrane. Dendritic spines are characterized by variety of shapes and sizes, differing greatly across brain areas, cell types, and animal species^1,2^. Learning and memory formation processes are tightly linked to remodeling or elimination of existing dendritic spines and outgrowth of new ones, what enable modulation of information transfer efficiency between neurons^1–4^. Additionally, changes in dendritic spines were detected after the various stimuli, such as drugs administration^5^, hypoxia^6^, environmental changes^7^, neurodevelopmental^8^, neurodegenerative^9^ and psychiatrics diseases^10^. For this reason, dendritic spines are believed to serve as sites for memory formation and storage, initiating memory consolidation through mechanisms of potentiation and depression of synaptic activity^11–14^. Dendritic spines shape is tightly linked with their function. For example, dendritic spines head size correlates with postsynaptic density area which one in turn reflect synaptic strength—the grater size the greater number of ion channels, kinases and other enzymes to effectively receive and response to synaptic input^15^. Dendritic spines neck geometry also appears to be important since it define how effectively electrical signal will transduce excitatory synaptic input from spine to dendrite^15,16^. For these reasons analysis and classification of dendritic spines shapes is used widely in many areas of basic and translational neuroscience, but the tools available to perform this kind of analysis are still quite limited.

The most widely used approach is classification of dendritic spines into predefined groups according to their key morphological features such as head and neck size. Traditionally these fixed classes are mushroom, thin, stubby, and sometimes filopodia. Mushroom spines have a large head and a small neck, separating them from a dendrite. They form strong synaptic connections, have the longest lifetime, and therefore are thought to be sites of long-term memory storage^12,17^. Thin spines have a structure similar to the mushroom spines, but their head is smaller relative to the neck. They are more dynamic then mushroom spines, and believed to be “learning spines”, responsible for forming new memories during synaptic plasticity process, accompanied by head enlargement^12,17^. Stubby spines typically do not have a neck. They are known to be the predominant type in early stages of postnatal development but are also still found in small amounts in adulthood, where they are likely formed due to disappearance of mushroom spines^18^. Filopodia are very long, thin dendritic membrane protrusions without a clear head, commonly observed in developing neurons and rare observed in mature neurons. Filopodia is thought to be the premature synapse, not yet having functional connection with the axon, therefore are usually excluded during synaptic density calculation. There are also additional spines shape classes which have been named by different research groups such as branched and cup-shaped spines^19^, but they are not widely used in the field.

Several software packages have been developed to segment and analyze dendritic spines, proposed approaches and their limitations have been reviewed in details^20,21^. Classification of dendritic spines into these predefined classes is the common approach to their shape analysis. The most widly used is Neuronstudio open source software that is based on manual selection of the spines by an operator^22^. Commercial Neurolucida software incorporated the decision tree classification algorithm from Neuronstudio^23^. Newly developed open-source software 3dSpAn offers 3 dimensional (3D) dendritic spines segmentation and classification using decision tree^24^. Supervised machine learning was previously used to classify spines into mushroom and non-mushroom groups^25^. Potential effectiveness of spines classification using semi-supervised learning was also reported^26^. An automated method of generating spine shape distribution based on 2D spine morphological features has been proposed^27^. However, most of these approaches have serious limitations, labor intensive and not openly available as we and others previously discussed^20,21^. Moreover, the commonly used classification approach itself has significant challenges^20,21^. It is very hard to define the clear border between mushroom, thin and stubby spine subtypes so such classification is often biased, subjective and poorly reproducible. Synaptic spines presents a continuum of shapes and sizes^15,28–32^ and an attempt to fit continuous distribution of spine shapes and sizes into pre-defined and rigid categories results in multiple sources of potential errors and information loss. Indeed, comparison of classification and direct morphometric measurement accuracy during dendritic spines morphology assessment demonstrated that the direct measurement approach is significantly more sensitive^33^.

To solve some of these issues we here present a novel approach to analyze synaptic spine shapes based on three dimensional experimental images. The proposed approach is based on application of chord length distribution histogram (CLDH) method, which utilizes a set of randomly selected chords within dendritic spine volume. The CLDH method has been previously applied in analysis of tumor morphology on MRI images^34^ but has never been used for neuroscience applications. This approach captures the spine morphology in an unbiased manner, and also allows to perform efficient and automated clustering of the spines. Importantly, the software that we developed is open-source and written in widely used Phyton language with GUI in Jupiter notebook. We hope that the proposed approach to unbiased and automated measurements and classification of synaptic spines will be adopted by the neuroscience community and serve as a useful resource for a variety of neuroscience research applications.

## Results

The developed analytical pipeline consists of 4 modules: confocal image segmentation, spine geometry feature extraction, spine classification and clustering. Each of these models will be described separately in the following sections. Detailed tutorial that describes installation and and instructions for use of this software package and dataset is provided as Supplement 2.

### Neuronal confocal image segmentation

**Figure 1:**
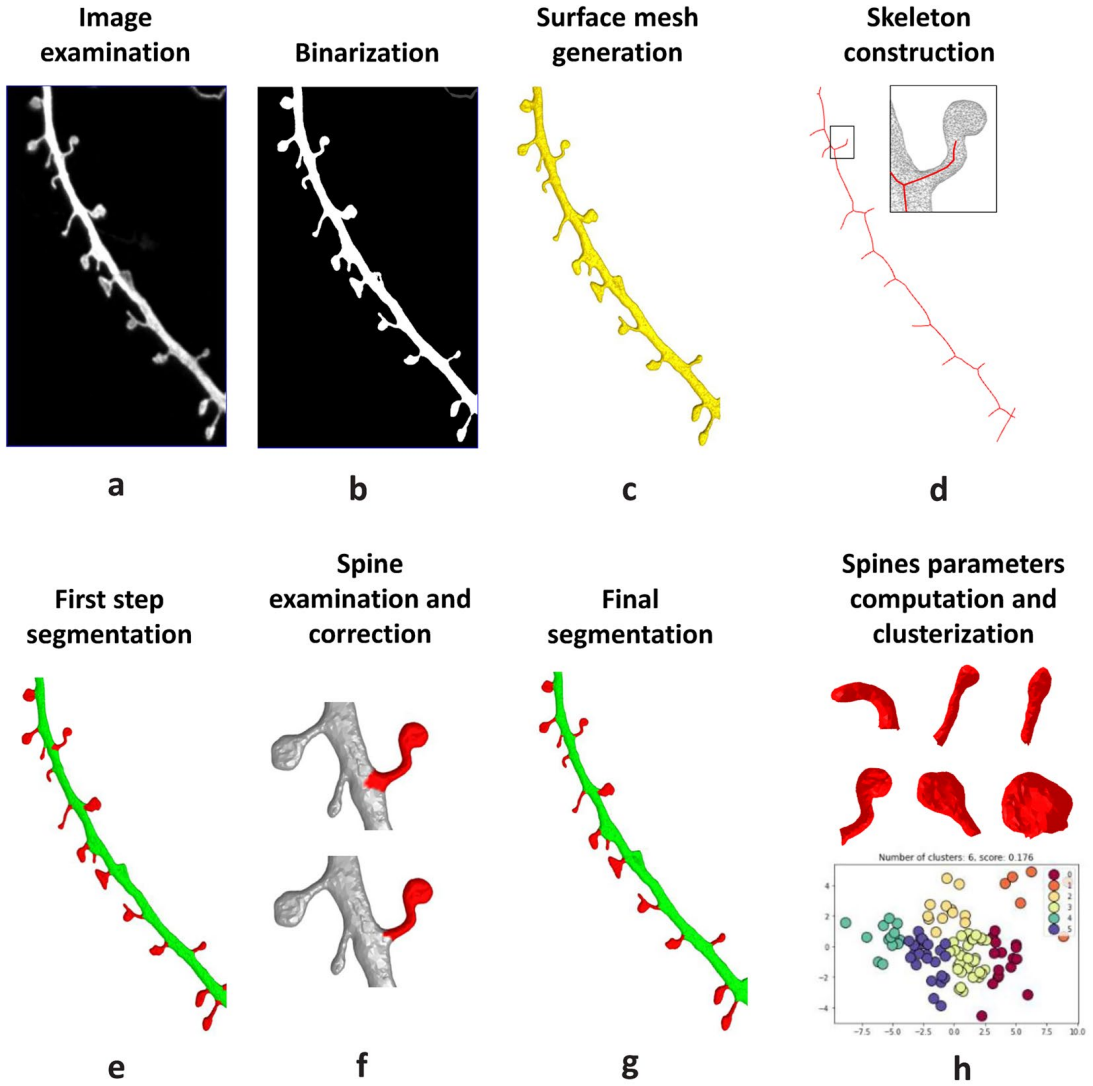
Python-based script pipeline to analyze dendritic spines morphology. (**a**) Three-dimensional high-resolution confocal dendrite image is uploaded and examine by the experimenter at the three axis x, y, z. (**b**) Image is binarized with help of adaptive thresholding algorithm. (**c**) Surface mesh to mark dendritic border is build. (**d**) Skeleton that invades dendrite protrusions is constricted inside the mesh. (**e**) Protrusions are labeled with red color at the dendrite, and (**f**) is examined manually at the step, where spine attachment is corrected and false positives are deleted if it is necessary, after that (**g**) segmented spines are saved. (**h**) Spines dataset is collected and various spines morphological parameters are calculated and extracted.

In our experiments primary mouse hippocampal cultures were transfected with EGFP-encoding plasmid at 8 days in vitro (DIV) and fixed at 15–16 DIV, when neurons became mature and develop extended processes and dendritic spines. We used the same approach extensively to study synaptic and neuronal morphology in our previous publications^35–39^. Neuronal image stacks of EGFP-positive cells were acquired by a conventional confocal laser scanning microscope with narrow pinhole 0.5 airy unit and 0.1 um z interval in order to achieve higher resolution to capture three-dimensional (3D) morphology of the spines. Existing segmentation solutions primarily focus on two-dimensional (2D) segmentation, processing only a 2D slice of the original 3D image, which may lead to significant loss of information about the shape of individual spines. To solve this problem we developed a new semi-automated 3D segmentation algorithm based on skeletonization, extending approach introduced previously for 2D images segmentation^40^. At the first step, the dendritic segment image (Fig. 1a) is binarized using adaptive thresholding algorithm (Fig. 1b), in which the threshold level is computed for each image pixel separately based on intensities of surrounding pixels^41^. When compared to global thresholding methods this approach is more sensitive to variations in dendritic spines morphology and intensity.

At the next step binarized dendrite image is converted to a polygonal mesh using Poisson surface reconstruction (Fig. 1c)^42^. Dendrite skeleton, represented as a set of line segments in 3D space, is constructed via Mean Curvature Skeleton algorithm^43^ (Fig. 1d) and each vertex of the dendrite mesh is labeled as either “spine” or “shaft” based on the dendrite skeleton structure and spatial position (Fig. 1e). User may correct initial segmentation parameters (Fig. 1e), then examine, check, and if necessary, exclude detected spines. Spine connection to the shaft can be manually adjusted using a wavefront algorithm to achieve better segmentation results (Figs. 1f and S1). After all segmentation procedures are completed (Fig. 1g), individual spine geometry is extracted as a set of polygonal meshes (Fig. 1h). Segmentation results of the same example image by 3 experts having small fluctuation with average variability (Fig. S2) of 3 selected spines features lies from 12,7% to 17,3%. Segmentation results of other public available example image with lower z axis resolution is shown on Fig. S3.

### Dendritic spines morphological feature extraction

**Figure 2:**
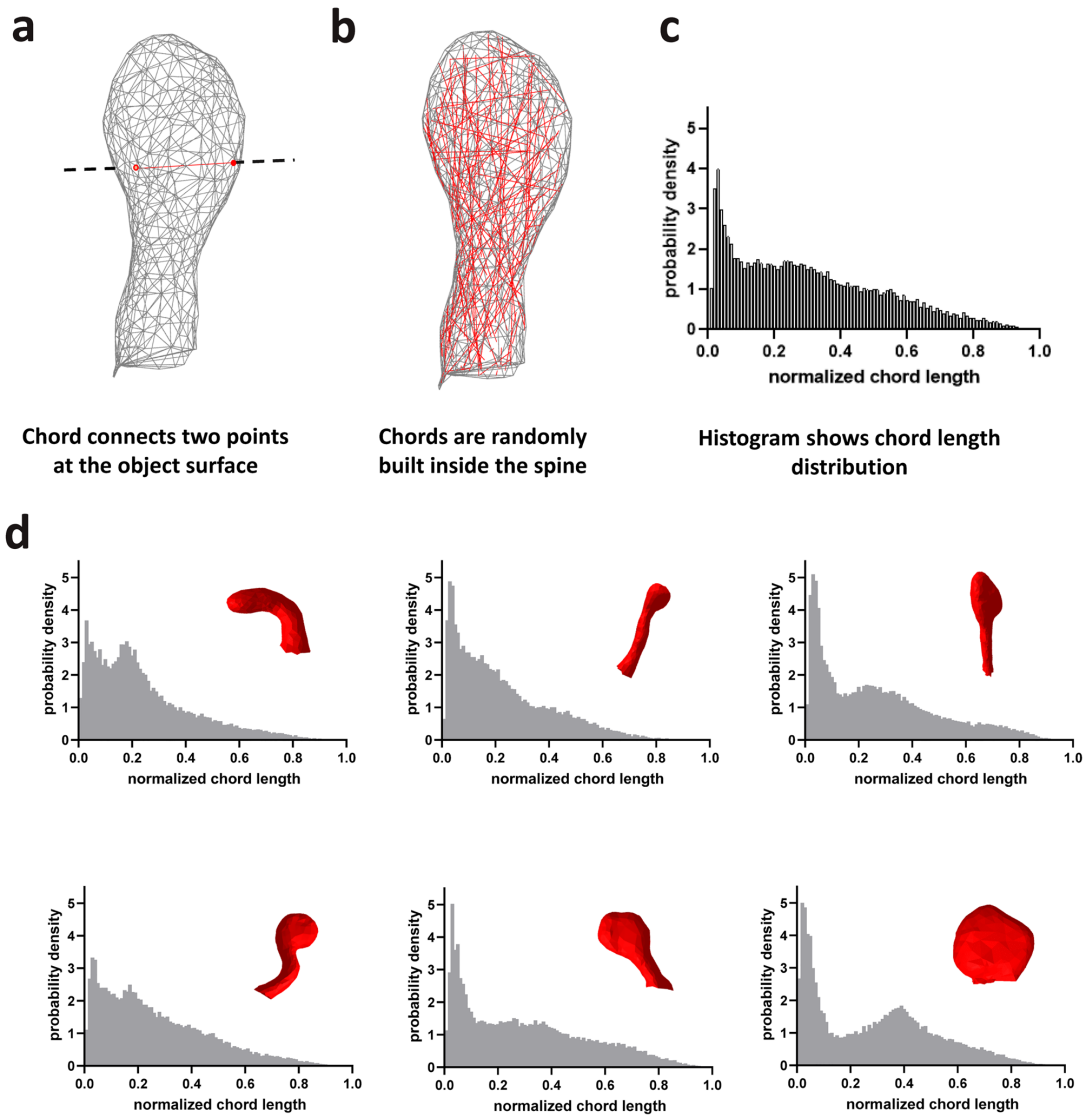
Dendritic spine shapes descriptor—chord length distribution histogram (CLDH). (**a**) Chord is a straight-line segment whose endpoints both lie on the spine surface. (**b**) Chords are randomly built inside the spine and (**c**) histogram characterizing their length distribution is built. (**d**) Examples of CLDH histograms for spines with various shapes with corresponding spines also shown.

After extracting individual spines as polygonal meshes, we calculate a set of numerical features for each spine in order to describe the spine shape and size. Based in previously published studies we formed a set of 11 basic or numerical features. We extracted 8 features introduced by^25^: spine length ($L$), volume ($V$), surface area ($S$), convex hull volume ($CHV$), convex hull ratio ($CHR$), average distance ($AD$), coefficient of variation in distance ($CVD$) and open angle ($OA$). We also adopted 3 additional features introduced by^44^: foot area ($FA$), length to volume ratio ($LVR$), and length to area ratio ($LVA$) for 3D dimensional case (see “Material and methods” section). In addition to the set of 11 basic features we introduce a more complex and qualitatively different measurement—chord length distribution histogram (CLDH). The CLDH method has been previously applied in analysis of tumor morphology^34^ but has never been used for neuroscience applications. Chord is a straight-line segment whose endpoints both lie on the spine surface (Fig. 2a). In this method, chords are randomly built inside the spine (Fig. 2b) and then distribution of generated set of chords lengths is represented as a histogram (Fig. 2c). Since the chords are built inside the spine randomly the histogram of their length distribution depends on their number and differs from trial to trial, but when number of chords is large enough these fluctuations become indistinguishable (Fig. S2). After n = 30,000 of chords inside the spine the Jensen-Shannon distance between histograms reaches the plateau, and it means that future increase in the chords number will not increase the CLDH accuracy (Fig. S4 B). Moreover, we count pairwise distance between histograms clearly showed that there are no spines with the same distribution in our dataset with n = 30,000 chords, since all values lies above zero (Fig. S4 C, D). As obvious from spines shapes examples, spines differ significantly in the form of corresponding CLDH (Fig. 2d). The advantage of this approach is that such distribution is invariant to translation and rotation of object’s geometry, and by normalizing chord lengths within a given object, this distribution can also be made invariant to object’s scale. This approach may also eliminate artificial biases introduced through assumptions about spine structure, like a subjective distinction between head and neck, etc. Thus, CLDH can provide a detailed problem-independent description of any given 3D spine shape. A disadvantage of CLDH approach is that this feature is surjective, which means that different spine shapes can produce similar distribution of chord lengths by chance.

### Spine classification using Support Vector Machine (SVM)

**Figure 3:**
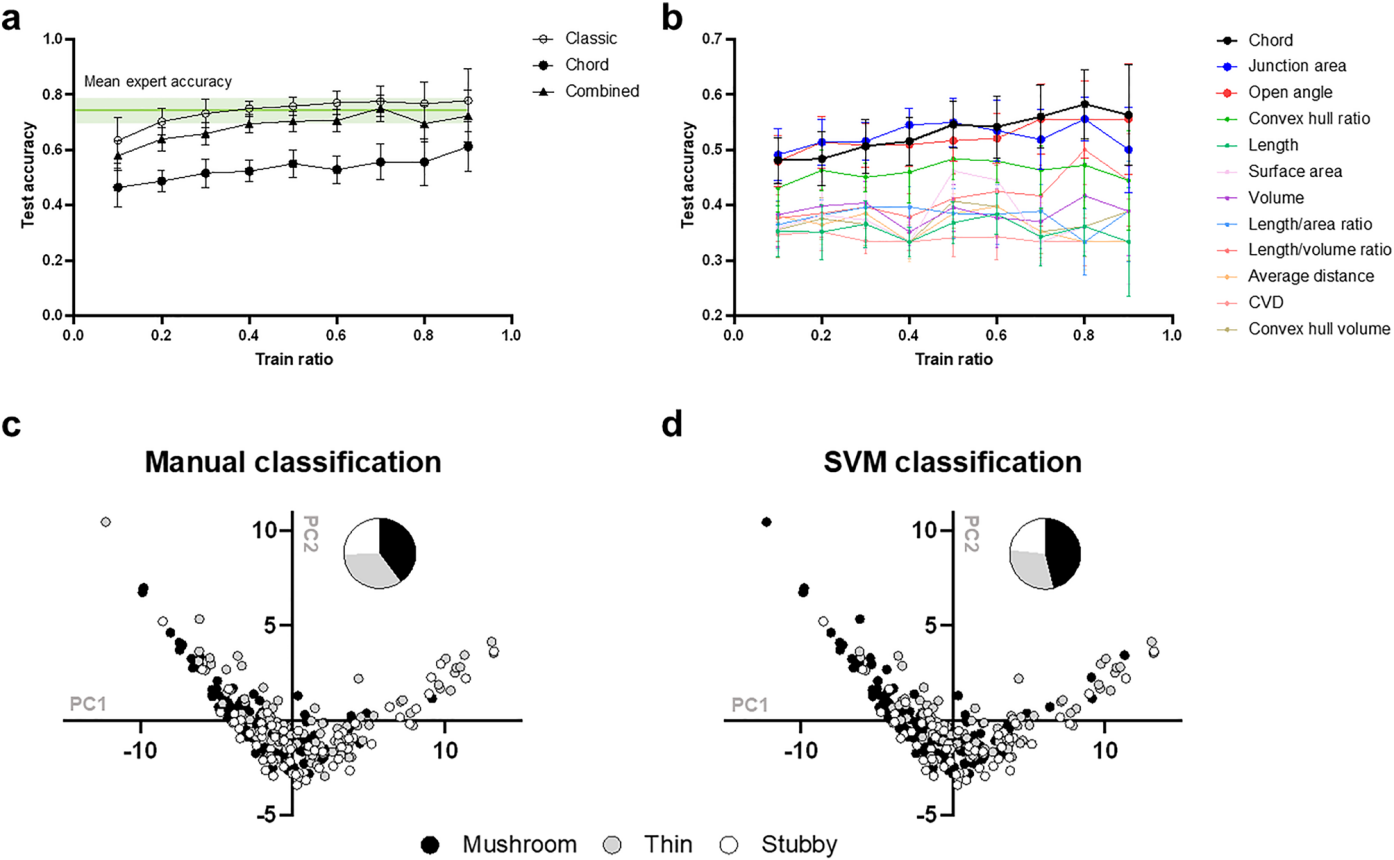
SVM-based dendritic spines classification. (**a**) Accuracy of SVM classification as a function of training dataset ratio for (**a**) classic metrics set (solid circles), CLDH metric (black circles) and their combination (black triangles) and (**b**) various classic metrics (colored circles) in comparison to CLDH (black circles) for n = 30 trials. Junction area and Open angle give the highest accuracy among classic features. Data shown as mean ± SD. (**c**,**d**) Spines classification map with classic metrics in two-dimensional principal components coordinates based on manual (**b**) and SVM (**c**) classification. For panels c and d mushroom spines are shown in black, thin in gray and stubby in white.

The metrics described above were used to analyze distribution of spine shapes in neurons. One of the most widely used approaches is to classify spines intro distinct morphological groups, such as mushroom, thin, and stubby spines. Classification of the spines into these categories is typically based on a simple decision tree and uses primitive morphological features, such as for example approach implemented in Neuronstudio^22^. To improve on this procedure, we developed a classification tool that uses machine learning algorithm and relies on more advanced spine shape descriptors as described in the previous section. To train the classifier, each spine from collected images was independently labeled by 8 experts familiar with neuronal imaging as either *mushroom*, *thin*, *stubby*, *filopodia* or *outlier* (Fig. S5). The baseline “consensus” classification was generated by assigning each spine the most common label among expert classifications (Fig. S5). We then removed outliers (no expert consensus has been reached) and filopodias (due to their small number in the dataset) (Fig. S5), and balanced dataset to equally represent all spine types. After building the baseline dataset based on experts consensus opinion, we trained the Support Vector Machine (SVM) to perform automatic classification. SVM attempted to separate spines into the classes in the feature space using hyperplanes, and a non-linear function (kernel) can be used to construct non-linear separators. We tested Radial Basis Function (RBF) kernel^45^, Histogram Intersection (HIS) kernel^46^, Laplasian Radial Basis Function kernel^47,48^ (Figs. S6 and S7) and linear kernel^49^ to classify data in classic/CLDH and combined feature space, where the last one showed the best results. Test accuracy plot (Fig. 3a) averaged for n = 30 trials proved the concept that SVM is trainable. The max accuracy is 0.77 (SD = 0.5) for classic, 0.55 (SD = 0.06) for CLDH and 0.75 (SD = 0.05) for combined metrics for 0.7. train ratio, with the larger training dataset the higher accuracy will be (Fig. 3a). SVM classification accuracy potentially achieving that of a human operator (0.77, n = 8, SD = 0.06), which was measured in the relation to the consensus (Fig. 3a), with classic and combined features. Accuracy for CLDH is lower.in comparison to classical features combination, so the accuracies when each of the 11 manual statistics is used as a one-dimensional variable for classification was measured (Fig. 3b). The highest accuracies comparable with CLDH were obtained for Junction area and Open angle, and lower for Convex hull ratio features, while for other features the accuracies lies above CLDH. The distribution of mushroom, thin and stubby spines obtained with manual (Fig. 3c) and SVM (Fig. 3d) in classic feature space classification are shown in two-dimensional coordinates built with help of Principle Component Analysis (PCA). Our results demonstrated that it is possible to simplify labor intensive and biased classification process using machine learning (Fig. 3). However, the disadvantage of this approach is that the training of this classifier still depends on the expert opinion. The expert bias can be partially offset by consensus procedures as used in our study, but in that case it requires many experts and the procedure becomes even more labor intensive. Also, we would like to notice that only 20% of spines in our dataset were assigned by experts to one class unanimously, and for 17% of spines it was impossible to get the consensus. Such kind of dataset ambiguity may complicate machine learning process. Moreover, classification-based learning procedure depends on particular data type and experimental conditions and must be repeated every time new dataset is analyzed. Nevertheless, once trained that algorithm may fast and efficiently classify spines during batch processing for example in preclinical drag screening tests as a redout of neuroprotective effect.

### Spine clustering using automated procedures

Despite the fact that classification into mushroom, thin, and stubby spines is widely used, it has numerous disadvantages and ever-increasing number of publications argue about the urgent need for more precise and unbiased clustering approaches as previously discussed by us and others^20,21^. In order to develop automated clustering procedure we considered two most common clustering algorithms—k-means method^50^ and DBSCAN (Density-Based Algorithm for Discovering Clusters)^51^. The k-means method seeks to minimize total intra-cluster variance of cluster points from the centers of these clusters. It allows to directly control the number of clusters into which the data is grouped by setting its adjustable parameter k—the number of clusters. The DBSCAN algorithm uses a set of points in multidimensional feature space and groups together points that are closely packed together (points with many close neighbors), marking as outliers points that lie in areas of low density (whose nearest neighbors are far away). DBSCAN’s main advantage is the ability to use various distance metrics that might be a better fit for the selected feature space, unlike k-means, that only operates using Euclidean distance. However, absence of direct control over the number of clusters and presence of “noise” points makes working with DBSCAN less intuitive and more difficult to interpret its output. In our studies DBSCAN clustering in classic feature space using Euclidian distance and DBSCAN clustering in CLDH feature space using Jensen–Shannon distance was attempted. However, in both cases very large number of clusters and outliers was generated (data not shown), making such clustering not informative.

**Figure 4:**
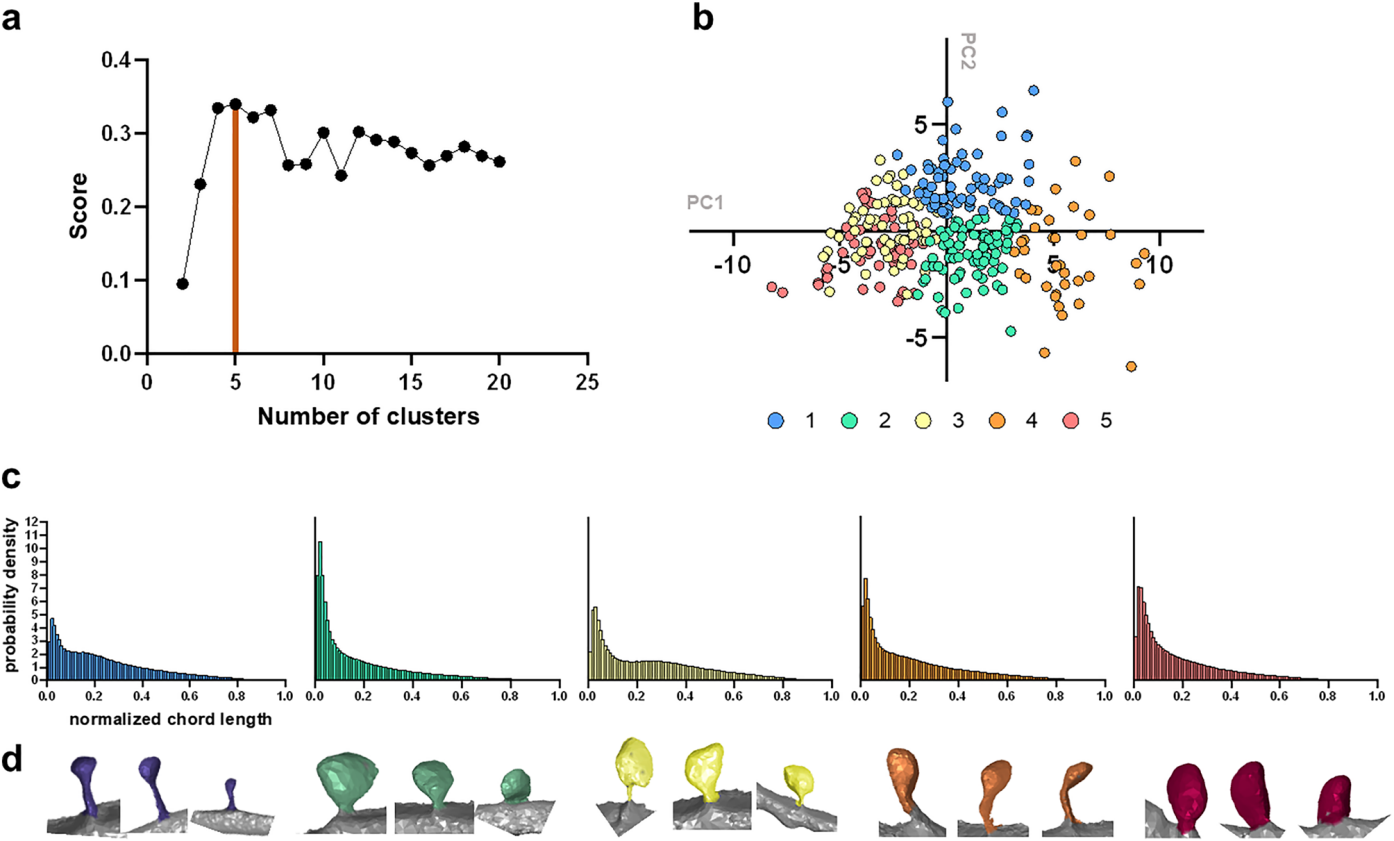
Dendritic spines clustering in CLDH feature space using max interclass divergence criteria. (**a**) Clustering coefficient score as a function of the cluster number. The max value for n = 5 is labeled with red line. (**b**) Clustering map in two-dimensional principal components coordinates, where clusters 1 to 5 are labeled with different colors as indicated. (**c**,**d**) CLDH histograms (**c**) and representative examples of spines (**d**) are shown for each cluster 1 to 5.

After failure of DBSCAN approach we also implemented k-means algorithm with algorithmically determined number of clusters. Besides using the classical elbow and silhouette score^52,53^ we proposed the new metric called “max class divergence criteria”. This metric is based on the assumption that clustering quality is better when clusters maximally differ from each other in the abundance of mushroom/thin/stubby classes, as predefined by experts. The advantage is that such an approach is considering the particular type of clustering data. Without such information assessing clustering quality is difficult. Clustering in CLDH feature space resulted in maximum 0.34 score of max class divergence criteria for n = 5 clusters (Fig. 4a). Clusters distribution is shown in two-dimensional coordinates on Fig. 4b. The mean graphs of CLDH histograms are shown for each class of spines on Fig. 4c with the spines examples of each cluster on Fig. 4d. The similarities in the shape of spines within each cluster are obvious (Fig. 4d). The average and std. deviation of classic features quantified for each cluster are shown on Fig. S8. In classic feature space max score was 0.31 for n = 4 clusters with PCA data dimensionality reduction (Fig. S10 A) and n = 14 without PCA (Fig. S10 C). Using elbow method resulted in n = 5 using CLDH as descriptor (Fig. S9) and n = 5 and n = 7 for classic metrics with (Fig. S10 E) and without PCA (Fig. S10 G), correspondingly. Silhouette method detects n = 5 as optimal cluster number at k-means clustering using CLDH feature (Fig. S9). Other method of number of clusters determination—GAP statistics^54,55^ doesn`t show appropriate result with such kind of data (data not shown)^54^. For some clustering result using t-SNE method will be better for visualization so it is added for user choice to the code. Notably, use of 3 independent metrics yielded 5 clusters in CLDH feature space clustering task (Figs. S4 and S9). In contrast, the same metrics in classic feature space resulted in highly variable number from n = 4 to n = 14 and content of clusters for the same clustering algorithm (Fig. S10). Thus, using CLDH metric provided more robust output of clustering task in our experiments.

### Comparison of spine classification and clustering outputs

**Figure 5:**
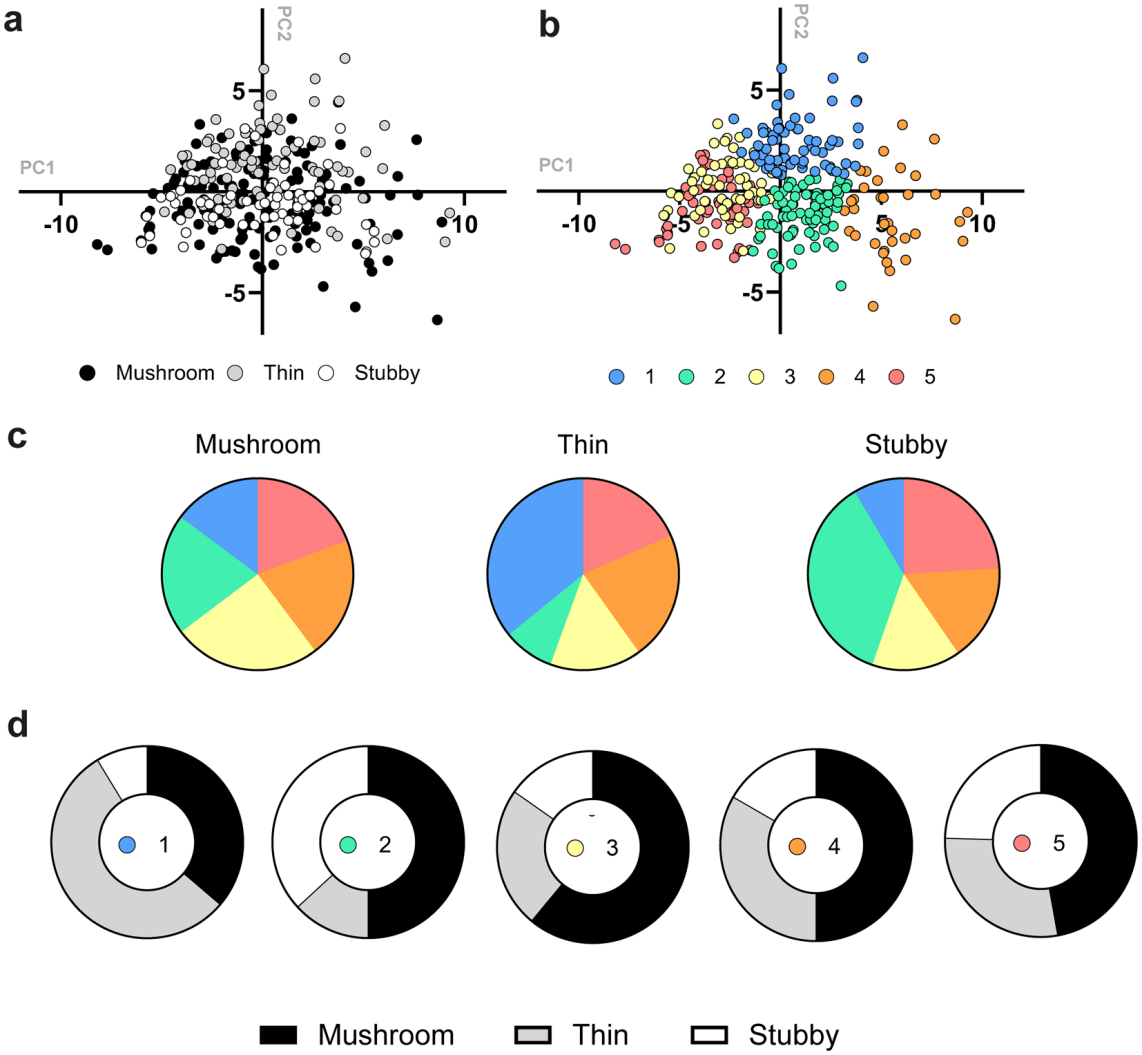
Comparison of dendritic spines classes and clusters. (**a**,**b**) Classification (**a**) and clustering (**b**) maps are built using two-dimensional principal components coordinates. (**c**) Enrichment of each cluster in mushroom, thin and stubby classes after normalization to total number of spines in each class is shown as a pie chart. (**d**) Enrichment of mushroom, thin and stubby classes in each cluster is shown as a pie chart. On panels a and d mushroom spines are shown in black, thin in gray and stubby in white. On panels b and c clusters 1 to 5 are labeled with different colors as indicated.

It is informative to compare results of spine classification and clustering for the same experimental dataset. The classification approach is based on 3 pre-determined classes of spines—mushroom, thin and stubby. The clustering approach resulted in 5 classes of spines in our analysis (cluster 1 to cluster 5) (Fig. 4). As a first step we represented results of classification and clustering as two-dimensional maps with principal component coordinates (Fig. 5a,b). As a next step we calculated relative enrichment of these classes in each other (adjusted for cluster size) (Fig. 5c,d). We concluded that with every proposed clustering method there was no clear conformity between classes and clusters, but there are some obvious trends. For example, clusters 1 and 2 content differ significantly between mushroom/thin/stubby classes, while clusters 3, 4, and 5 are equally represented in all 3 classes (Fig. 5c). It also appears that for mushroom spines clusters 2 and 3 are most common, for thin spines clusters 1 and 4 are more common, and for stubby spines clusters 2 and 5 are most common (Fig. 5c). These conclusions are in agreement with difference in spine shapes as presented on examples on Fig. 4d.

## Discussion

### CLDH method is a powerful non-numerical feature for dendritic spines shape description

We introduced a qualitatively new feature for spine morphology description—chord length distribution histogram ($CLDH$). In this method, chords are randomly built inside the spine and then a distribution of generated set of chords lengths is represented as a histogram. The CLDH method has been previously applied in analysis of tumor morphology^34^ but has never been used for neuroscience applications. Interestingly, only this feature was sufficient to obtain clearly separated clusters with common shapes in spines dataset clusterization task (Fig. 4b), while using 10 classical numerical features from^25^ and^44^ yielded lower clustering quality metrics value (Fig. S10A, C).Intriguingly, CLDH give more stable results in clustering giving the same number of clusters using various metrics rather than classic metrics set (Figs. 4b, S9), where the number of clusters varies greatly between them To explain these findings, we propose that information content within chord histogram is higher than in the limited set of numerical features,therefore CLDH is a more precise descriptor of spine shape and is suitable for spines clustering according to their shape. Our results suggest that further development of qualitatively new metrics and their combinations might provide better tools for spine shapes analysis.

### Use of machine-learning classification for synaptic spine analysis

Supervised and semi-supervised machine learning algorithms have been used previously to classify spines in some success^25,25,56^. Kashiwagi et al.^25^ classify spines into only two groups: mushroom and non-mushroom. Shi et al.^26^ noticed that using three groups instead of two give much high error rates, with stubby spines are the dominant source of the errors. Ghani et al. offered statistical machine learning-based approach to two-photon microscopy 2D spines images classification^27^. In our experiments max accuracy achieved with SVM method is comparable with average human expert accuracy, which is 77% (n = 8, SD = 0.06) (Fig. 3a). The higher accuracy rates have been reported in semi-supervised approach with less training datasets^57^, but these authors use only one expert, so it is impossible to assess the accuracy in relation to human operator. In our study we utilized “consensus approach” based on opinion of 8 independent experts (Fig. S5). Using this approach, we demonstrated that SVM approach is potentially achievable to a human operator consensus in the classification task with classic and combined features (Fig. 3). Classification in CLDH feature space give lower than experts accuracies. One possible reason is that CLDH is high dimension metric that represents dendritic spine shape as a vector of 100 values. Data models with such many parameters could be complicated for classification task and therefore more data required for classifier training.

However, clustering can find hidden dependencies in the data which are not detected by classifier and dimension reduction techniques^58^, and sometimes the pre-clustered data can show higher classification accuracy^59^, which may be used to overcome this issue. SVM accuracy in CLDH feature space is comparable to Junction area, Open angle metrics, to Convex hull ratio in lower extend, and higher than other classic metrics when they used as one-dimensional variables to train SVM classificator. From these observations we may assume that CLDH contain valuable information about dendritic spine shape. Notably, the mentioned above metrics, firstly used in^25^, are describing spines geometry in more complex way rather than often intuitively used length, volume and etc., which points that more sophisticated descriptors showing better result to characterize dendritic spines shape.

The output of SVM depends on selected features of the training vector, in agreement with the previous findings^26^. Using SVM approach may be useful in high throughput spine analysis experiments, such as for example drug screening in neurodegenerative disease mouse models as for example our laboratory routinely performs^35–39,60–62^. Indeed, after initial efforts to train SVM the results can be obtained in just in a few seconds (Fig. 3). This approach can be enhanced in the future by defining more informative features for SVM learning and improving learning algorithms.

### Clustering provides evidence for existence of multiple classes of spines

Unbiased spine clustering experiments performed by us (Fig. 4) and by previous investigators^25,44,63^ always suggested existence of multiple classes of spines that do not neatly match with mushroom/thin/stubby classification (Fig. 5). As we and others discussed previously^20,21^, spine shapes and sizes represent continuum and not discrete categories. Different classes labeled by experts consensus are consist of various clusters and include each of them (Fig. 5c). There is some enrichment of mushroom, thin and stubby spines to different clusters, but all of these classes are present in each cluster (Fig. 5d). Different clustering approaches produce different clusters, and it is difficult to assess the quality of the clustering and reliable criteria to choose one of them. This is a major obstacle for using clustering for spine analysis. To address this problem we offered a clustering quality assessment where expert opinion is taken into account—max class divergence criteria. Max class divergence criteria is based on the assumption that the better clustering quality is achieved when clusters are most differ from each other in terms of mushroom/ thin/stubby classes content in each class. Application of max class divergence criteria in combination with CLDH metric enabled us to achieve robust clustering results (Figs. 4 and 5). In the future biological relevance of different spine clusters needs to be established by comparing biochemical and functional properties of different clusters.

## Conclusions

We developed an open-source spine analysis software that provides robust tool for 3D dendritic spine analysis using classification and clustering approaches with various numerical shape descriptors and introduced new one—chord length distribution histogram (CLDH). The source files for image segmentation, feature extraction, classification,clustering procedures, and spines segmentation and spines expert classification dataset, are available via a public repository. The tutorial for installing and using the software is provided as Supplementary 2. The software is written in Python language. Python is currently the most widely used multi-purpose, high-level programming language, and if necessary, software code can be edited by the user as needed. Moreover, recently were developed a library for integrating Python and ImageJ—intensively used by numerous scientists wide purpose images processing software^64^. We hope that this software will be adopted by the neuroscience community and serve as a useful resource for a variety of neuroscience and neurodegenerative research applications.

## Methods

### Primary hippocampal neuronal cultures

All animal procedures were approved by the Bioethics Committee of the Peter the Great St. Petersburg Polytechnic University at St. Petersburg, Russia and followed the principles of European convention (Strasbourg, 1986) and the Declaration of International medical association about humane treatment of animals (Helsinki, 1996). Primary hippocampal neuronal cultures of dissociated hippocampal cells were prepared from newborn FVB background mice as we previously described^35–39^. Briefly, the hippocampus of postnatal day 0–1 mouse pups were digested with papain solution (30 min at 37 °C; Worthington, #3176), then dissociated with 5 mg/ml Deoxyribonuclease I (Macherey Nagel GMBH, #R1542S) solution. Neurons were plated in a 24-well culture plate on 12 mm glass coverslips precoated with 1% poly-D-lysine (Sigma, #p-7886) in Neurobasal-A (Gibco, #10888022) medium supplemented with 2% B27 (Gibco, #17504044), 1% heat-inactivated fetal bovine serum (FBS, Gibco, #10500064), 0.5 mM L-Glutamine (Gibco, #25030024) and maintained at 37 °C in a 5% CO2 incubator at 24-well glass plate.

### Data acquisition

Transfection of primary hippocampal neurons is performed at 6–7 DIV, at 15–16 DIV, when hippocampal neurons reach maturity and form extensive synaptic contacts cells are fixed with a solution of 4% formaldehyde and 4% sucrose in PBS, pH 7.4 for 15 min and then extensively washed with PBS to remove fixation solution. Transfection was performed with calcium transfection kit was purchased from Clontech (#631312) with pLV-eGFP (Addgene, #36083). To enhance fluorescent signal neurons were IHC stained with anti-GFP antibodies at 1/300 dilution (Invitrogen, #A-11122) overnight +4 C and anti-rabbit Alexa-Fluor 488 (Invitrogen, #R37116) antibodies for 2 h at room temperature, then glasses were mounted with ProLong™ Glass Antifade Mountant (ThermoFisher, #P36980). For assessment of dendritic spines morphology, a Z-stack of the optical section was captured with a confocal microscope (Thorlabs). For dendritic analysis 2048 × 2048 pixels images with 0.025 μm/pixel resolution were captured with Z interval of 0.1 μm using a 100 × objective lens (NA = 1.4, UPlanSApo; Olympus) with 0.5 AU to achieve the best resolution.

### Spine geometry extraction

In this section we describe a semi-automated algorithm used to extract individual spine geometry from 3D voxel image data. We propose a new semi-automated algorithm based on approaches from previously published 2D and 3D algorithms. The algorithm can be broadly separated into 3 steps: constructing a 3D polygonal mesh of the dendritic segment’s surface; segmenting the surface mesh into dendrite and spines areas; and extracting areas marked as spines into individual spine meshes. Totally 275 spines were segmented from n = 54 cropped dendritic images.

#### Surface mesh construction

Surface mesh construction is performed in two steps: voxel image binarization and 3D surface construction. A simple thresholding algorithm for image binarization produces poor results because of the varying intensity values withing the confocal cultured neurons images. The problem is especially acute when binarizing low-intensity spine necks.

As such, a combination of simple and adaptive thresholding algorithms is used. Threshold value for the voxel with coordinates $x, y,z$ is calculated as:

$$Thres\left(x,y,z\right)=BaseThres\cdot \left(1-t\right)+t\cdot LocalThres\left(x,y,z\right),$$

where $BaseThres$ is a user-defined constant threshold, $LocalThres(x,y,z)$ is the mean intensity of voxel in a window around target voxel, $t$ is a user-defined constant in range $[0, 1]$.

The polygonal surface mesh is constructed using the Poisson Surface Reconstruction method^42^. The polygonal mesh produced by the Poisson method is smooth, closed, and triangulated. The method takes a set of points belonging to the reconstructed surface and normal vectors in those points as input. Surface points are extracted as the difference between binarized image and its binary erosion. Normal vectors are extracted by calculating binary image gradient along each axis.

#### Surface mesh segmentation

Each surface mesh vertex needs to be marked as either belonging to the dendrite shaft or a spine. Surface mesh segmentation algorithm is based on the skeleton graph algorithm from^40^ extended into 3D space and cylinder fitting approach from^25^.

Firstly, surface mesh skeleton is constructed using the Mean Curvature Skeleton algorithm^43^ that extracts a curve skeleton from a triangulated surface mesh without borders by iteratively contracting the input triangulated surface mesh. The resulting skeleton is an undirected graph $G$, where each skeleton vertex has an associated 3D coordinate, and every surface mesh vertex can be mapped onto a singular skeleton vertex that it contracted to.

We then find the skeleton subgraph ${G}_{D}\subset G$ corresponding to the dendrite shaft. We assume that the image contains only one dendrite shaft. A reduced graph ${G}^{*}$ is constructed by consequently replacing all vertices with degree of 2 with an edge. Reduced graph ${G}^{*}$ is acyclic and consists of leaf and junction vertices only. Any subgraph ${G}_{i}\subset G$ has an equivalent subgraph ${G}_{i}^{*}\subset {G}^{*}$ that it can be reduced to. Longest vertex-wise paths ${P}_{1}, .., {P}_{m}\subset {G}^{*}$ are calculated. Assuming that the dendrite shaft segmented is relatively linear while spines are positioned at an angle to the dendrite shaft, we can calculate sum path angle $\alpha \left({P}_{j}={v}_{1}..{v}_{n}\right)={\sum }_{i=2}^{n-1}\alpha \left(\overrightarrow{{v}_{i-1}{v}_{i}},\overrightarrow{{v}_{i}{v}_{i+1}}\right)$ for each path ${P}_{j}$ and choose the path ${P}^{*}=\mathrm{arg}\underset{\mathrm{j}}{\mathrm{min}}\alpha ({P}_{j})$ as the one corresponding to the dendrite shaft. Dendrite shaft skeleton subgraph ${G}_{D}\subset G$ is then selected as subgraph equivalent to ${P}^{*}\subset {G}^{*}$. Surface mesh vertices, mapped onto the vertices of dendrite skeleton subgraph ${G}_{D}$, are marked as belonging to the dendrite. Other vertices are marked as belonging to a spine.

However, due to the nature of the skeletonization algorithm in a three-dimensional case, as opposed to a two-dimensional described in^40^, a number of smaller spines does not have a dedicated skeleton branch and is also mapped onto the dendrite shaft subgraph, being falsely marked as belonging to the dendrite. To counteract this issue, an additional step, loosely based on the cylinder fitting method from^25^ is proposed. Surface vertices are thresholded based on their distance to the dendrite skeleton subgraph. Vertices closer than a selected threshold value are marked as belonging to the dendrite, while further vertices are marked as belonging to a spine. User is provided with a *SENSITIVITY* tuning parameter with a [0, 1] value range. Distance to skeleton is calculated for every surface vertex that is mapped onto the dendrite skeleton subgraph ${G}_{D}$. Threshold value is selected as *SENSITIVITY*th quantile of this distance distribution.

#### Spine mesh extraction

After each vertex has been marked as either “dendrite” of “spine”, individual spine meshes are extracted. Using a representation of the surface mesh as a graph, individual spines are separated from each other by finding connected components of vertices marked as “spine”. Each connected component is its own spine.

For each spine we then remove all the facets that don’t belong to that spine (every vertex of the facet must belong to this spine). We now have a surface mesh of the spine, however, there is now a hole in the mesh where the spine base was connected to the dendritic shaft. A simple fan triangulation algorithm is used to create triangulated “patches” over these holes, making the surface mesh closed.

### Feature extraction

In this section we describe the features extracted from individual dendritic spines, aimed at numerically representing the spines morphology. We extracted some of the most often used features and introduce a new feature—chord length distribution histogram.

#### Morphological features

Some of the most often used numerical features have been selected: we’ve extracted 8 features introduced in^25^ (spine length, volume, surface area, convex hull volume, convex hull ratio, average distance, coefficient of variance in distance, and open angle), as well as adapted some of the 2D features introduced in^44^ to 3D space (length to volume ratio, length to surface area ratio, foot area).

Several metrics use the value $\overrightarrow{c}$—centroid of the triangulated “patch” that fills the hole on the spine mesh where the spine was separated from the dendritic shaft.

Spine length ($L$) is calculated as:

$$L=\frac{1}{n}\sum\limits_{i=1}^{n}\left|\overrightarrow{{p}_{i}}-\overrightarrow{c}\right|,$$

where $\overrightarrow{{p}_{i}}$ are the vertices that are further from ${c}_{i}$ then 95% of the other surface vertices.

Average distance ($AD$) is calculated as:

$$AD=\frac{1}{N}\sum\limits_{j=1}^{N}\left|\overrightarrow{{p}_{j}}-\overrightarrow{c}\right|,$$

where $\overrightarrow{{p}_{j}}$ are surface vertices. Coefficient of variation in distance ($CVD$) is calculated as the coefficient of variation in $\left|\overrightarrow{{p}_{j}}-\overrightarrow{c}\right|$.

Open angle ($OA$) is calculated as:

$$OA=\frac{1}{N}\sum\limits_{i=1}^{N}\alpha \left(\overrightarrow{{p}_{i}}, \frac{1}{N}\sum\limits_{j=1}^{N}\left(\overrightarrow{{p}_{i}}-\overrightarrow{c}\right)\right),$$

where $\alpha (\overrightarrow{x}, \overrightarrow{y})$ is the angle between vectors $\overrightarrow{x}$ and $\overrightarrow{y}$ in range $[-\pi ,\pi ]$.

Convex hull ratio $(CHR)$ is calculated as:

$$CHR=\frac{CHV-V}{V},$$

where $CHV$ is convex hull volume, $V$ is spine mesh volume.

Foot area (FA) is calculated as the area of the triangulated “patch”.

#### Chord length distribution histogram

We introduce a numerical feature that attempts to summarize the shape of a dendritic spine—chord length distribution histogram (CLDH). It is a histogram-summarized distribution of lengths of chords within a given object. The advantage of this approach is that such distribution is invariant to translation and rotation of object’s geometry and by normalizing chord lengths within a given object, this distribution can also be made invariant to object’s scale. A disadvantage of this approach is that this feature is surjective, meaning different shapes might produce the same distribution of chord lengths. Storing and comparing distributions in their entirety for each spine is computationally expensive, so the distributions are summarized in histogram form.

Given that the number of possible chords within an object is infinite, the distribution is approximated using a Monte Carlo method. Pairs of random points on the mesh surface are chosen and a ray is cast from outside the mesh such that both points lie on the ray. The ray is intersected with the mesh, and distances between consecutive intersection points are used to calculate chord lengths. The process is continued until a set number of rays is cast.

### SVM classification

The dataset was manually classified by 8 experts into 5 classes (thin, mushroom, stubby, filopodia, outlier). Individual spines were classified into different classes by different experts, so a unified classification was constructed: each spine was assigned to a class that most experts assigned it to. When no clear consensus was reached as multiple classes received an equal highest number of expert “votes”, the spine was classified as an outlier. For the purposes of SVM classification we removed outlier spines from the dataset, as well filopodia spines, as there were only 6 of them in the dataset.

Using unified expert-produced classification we trained an SVM (Support Vector Machine) to perform automatic classification. SVM attempts to separate the classes in feature space using hyperplanes. In addition, a non-linear function (kernel) can be used to construct non-linear separators between classes. A commonly used kernel is Radial Basis Function (RBF):

$$RBF\left(x,{x}^{\mathrm{^{\prime}}}\right)=\mathrm{exp}\left(-\frac{{\Vert x-{x}^{\mathrm{^{\prime}}}\Vert }^{2}}{2{\sigma }^{2}}\right).$$

To train the SVM we constructed randomized spine subsets of various sizes. We call the size of a training dataset relative to the size of the entire dataset as *training dataset ratio*. When constructing training subsets class membership percentages (percentage of mushroom/stubby/thin spines) within the training subset was kept equal to the original dataset. From the remaining spines not included in the training dataset a testing dataset was formed. We constructed training datasets for training dataset ratios of $[0.1, 0.2,\dots , 0.9]$, used them to train SVMs in classic, CLDH and combined feature spaces, and evaluated their accuracy on corresponding testing datasets (Fig. 3a).

### Clustering

While most research so far has been focused on solving the classification problem of assigning spines to pre-determined classes (stubby, mushroom, thin, filopodia), the consensus has not yet been reached about the number of or meaningful biological distinction between these classes. The opinion of which class a given spine belongs to often differs between multiple experts, and even the same expert at different times^25^. Algorithms aimed at solving the classification problem require large manually annotated training datasets, that are time-consuming to produce and invariably carry within themselves the biases of a particular human expert responsible for data annotation. As such, a different approach has been introduced in^44^—trying to instead solve a clustering problem, wherein the data is divided not into pre-determined classes, but into an algorithmically-determined clusters. By doing so, we attempt to find new, data-driven spine groupings that don’t require a time-consuming process of manual annotation and are unaffected by human expert biases. In this section we describe various approaches to data clustering that were evaluated on the extracted feature dataset.

#### Clustering quality evaluation

Choosing an approach for evaluating clusterization quality is an unclear and problem-dependent task. One of the approaches is manual evaluation by a human expert; however, it is a very time- and resource-consuming process. To automate the evaluation process, we use numerical scores, also known as “internal” evaluation, although it should be noted that the resulting scores do not necessarily directly translate into the practicality of using said clustering.

We introduce a new approach to clustering quality evaluation based on expert classification. Given ${\mathbb{X}}=\left\{{X}_{1}, \dots , {X}_{m}\right\}$—clustering of spines into $m$ clusters and ${\mathbb{Y}}=\left\{{Y}_{1}, \dots , {Y}_{n}\right\}$—classification of spines into $n$ classes, we can calculate a matrix of distribution of classes over clusters.

$$M=\left(\begin{array}{ccc}\frac{\left|{X}_{1}\cap {Y}_{1}\right|}{\left|{X}_{1}\right|}& \cdots & \frac{\left|{X}_{1}\cap {Y}_{n}\right|}{\left|{X}_{1}\right|}\\ \vdots & \ddots & \vdots \\ \frac{\left|{X}_{m}\cap {Y}_{1}\right|}{\left|{X}_{m}\right|}& \cdots & \frac{\left|{X}_{m}\cap {Y}_{n}\right|}{\left|{X}_{m}\right|}\end{array}\right).$$

Note that each row can be viewed as a histogram of distribution of classes ${Y}_{1}, \dots , {Y}_{n}$ within cluster ${X}_{i}$ and ${\sum }_{j=1}^{n}{M}_{ij}=1$. The more different those histograms are from each other, the more different is the make up each cluster in terms of types of spines it contains. We can thus calculate mean distance between rows of $M$ and use it as a numerical score to evaluate clustering quality.

#### Clustering algorithms

In this paper we evaluate two of the more popular crisp clustering algorithms: k-means and DBSCAN.

The k-means method seeks to minimize total intra-cluster variance of cluster points from the centers of these clusters. It allows to directly control the number of clusters into which the data is grouped by setting its adjustable parameter k—the number of clusters.

The DBSCAN algorithm is density based—given a set of points in multidimensional feature space, the algorithm groups together points that are closely packed together (points with many close neighbors), marking as outliers points that lie in areas of low density (whose nearest neighbors are far away). The primary adjustable parameters of DBSCAN are $\varepsilon$—the maximum distance at which points can be considered close neighbors, and the distance metric between points in the multidimensional feature space.

The advantage of k-means is direct control over the number of clusters. DBSCAN’s main advantage is the ability to use various distance metrics that might be a better fit for the selected feature space, unlike k-means, that only operates using Euclidean distance. However, no direct control over the number of clusters and presence of “noise” points make it less intuitive to use DBSCAN and interpret its results.

To find best parameter values we experimented with3 approaches: elbow method, silhouette and maximizing quality function value. The elbow method is common technique for choosing optimal clustering parameter value. The method utilizes some algorithm-specific function $\varphi$ of clustering parameter that approaches zero as parameter value is increased. Optimal parameter value is determined by plotting function $\varphi$ and selecting a point of maximum graph curvature.

For k-means algorithm $\varphi (k)$ is the within cluster Sum of Squares function:

$$WSS=\sum \limits_{i=1}^{k}\sum \limits_{x\in {X}_{i}}{\left(x-{c}_{i}\right)}^{2},$$

where ${X}_{1}..{X}_{k}$ is the set of clusters, $\overrightarrow{{c}_{i}}$ is the centroid of the i-th cluster.

For DBSCAN algorithm $\varphi (\varepsilon )$ is number of points such that the distance to their 3-nearest neighbor is greater than $\varepsilon$.

The silhouette coefficient $s$^53^ is calculated using the mean intra-cluster distance (a) and the mean nearest-cluster distance (b) for each sample:

$$a=\frac{{\sum }_{i=0}^{k}\underset{{{x}_{i},\mathit{ }{x}_{j}\in c}_{i}}{\mathrm{max}}dist({x}_{i}, {x}_{j})}{k}$$

$$b=\frac{{\sum }_{i=0}^{k}{\mathrm{min}}_{j=0}^{k}\underset{{\mathrm{x}}_{\mathrm{i}}\in {c}_{i}, {x}_{j}\in {c}_{j}}{\mathrm{min}}dist({x}_{i}, {x}_{j})}{k}$$

$$s=\frac{b-a}{\mathrm{max}\left\{a, b\right\}},$$

where $k$ is number of clusters, ${c}_{i}$ is cluster.

#### Feature space

We apply k-means and DBSCAN clustering algorithms separately in feature spaces of classic features and CLDH. DBSCAN algorithm allow for use of different distance metrics. For classic features we only use Euclidean distance, while for CLDH we also experimented with applying a distance metric specifically designed to measure distance between distributions—Jensen–Shannon distance:

$$JSD\left(P, Q\right)=\sqrt{\frac{1}{2}D\left(P,\frac{1}{2}\left(P+Q\right)\right)+\frac{1}{2}D\left(Q, \frac{1}{2}\left(P+Q\right)\right)},$$

where $P, Q$ are histogram vectors for two spines, $D$ is Kullback–Leibler divergence:

$$D\left(P, Q\right)={\sum }_{i}{P}_{i}\mathrm{log}\left(\frac{{P}_{i}}{{Q}_{i}}\right).$$

### Ethics approval and consent to participate

All experimental protocols were approved by the Bioethics Committee of the Peter the Great St. Petersburg Polytechnic University at St. Petersburg, Russia and followed the principles of European convention (Strasbourg, 1986) and the Declaration of International medical association about humane treatment of animals (Helsinki, 1996). All methods were carried out in accordance with relevant guidelines and regulations. The study was carried out in compliance with the ARRIVE guidelines.

### Supplementary Information

Supplementary Figures.

Supplementary Information.

## Data Availability

All original code, example image and dataset consisting of segmented dendritic spines meshes, experts classification and experts consensus labeling has been deposited at GitHub and is publicly available as of the date of publication at https://github.com/spbstu-applied-math/SpineTool.Software tutorial and dataset description are provided at Supplementary 2.

## Materials availability

This study did not generate new unique reagents.
