# Author Correction: SpineTool is an open-source software for analysis of morphology of dendritic spines

**DOI:** 10.1038/s41598-024-54127-4

**Published:** 2024-02-19

**Authors:** Ekaterina Pchitskaya, Peter Vasiliev, Daria Smirnova, Vyacheslav Chukanov, Ilya Bezprozvanny

**Affiliations:** 1 Laboratory of Molecular Neurodegeneration, Peter the Great St. Petersburg Polytechnic University, Khlopina St. 11, St. Petersburg, Russia 194021; 2 Department of Applied Mathematics, Peter the Great St. Petersburg Polytechnic University, Polytechnicheskaya St. 29, St. Petersburg, Russia 195251; 3 Department of Physiology, UT Southwestern Medical Center at Dallas, Dallas, TX 75390 USA

Correction to: *Scientific Reports* 10.1038/s41598-023-37406-4, published online 29 June 2023

The original version of this Article contained an error, where the names of the authors Ekaterina Pchitskaya, Peter Vasiliev, Vyacheslav Chukanov and Ilya Bezprozvanny were incorrectly given as Pchitskaya Ekaterina, Vasiliev Peter, Chukanov Vyacheslav and Bezprozvanny Ilya.

The original Article has been corrected.

