# Supplementary material for: SpineTool is an open-source software for analysis of morphology of dendritic spines
Source: Sci Rep. 2023 Jun 29;13:10561. doi: 10.1038/s41598-023-37406-4 (PMC10310755; doi:10.1038/s41598-023-37406-4)
Supplement: Supplementary file 1 — Supplementary Figures. [file 41598_2023_37406_MOESM1_ESM.pdf]

Supplementary 1 – Supplementary Figures.

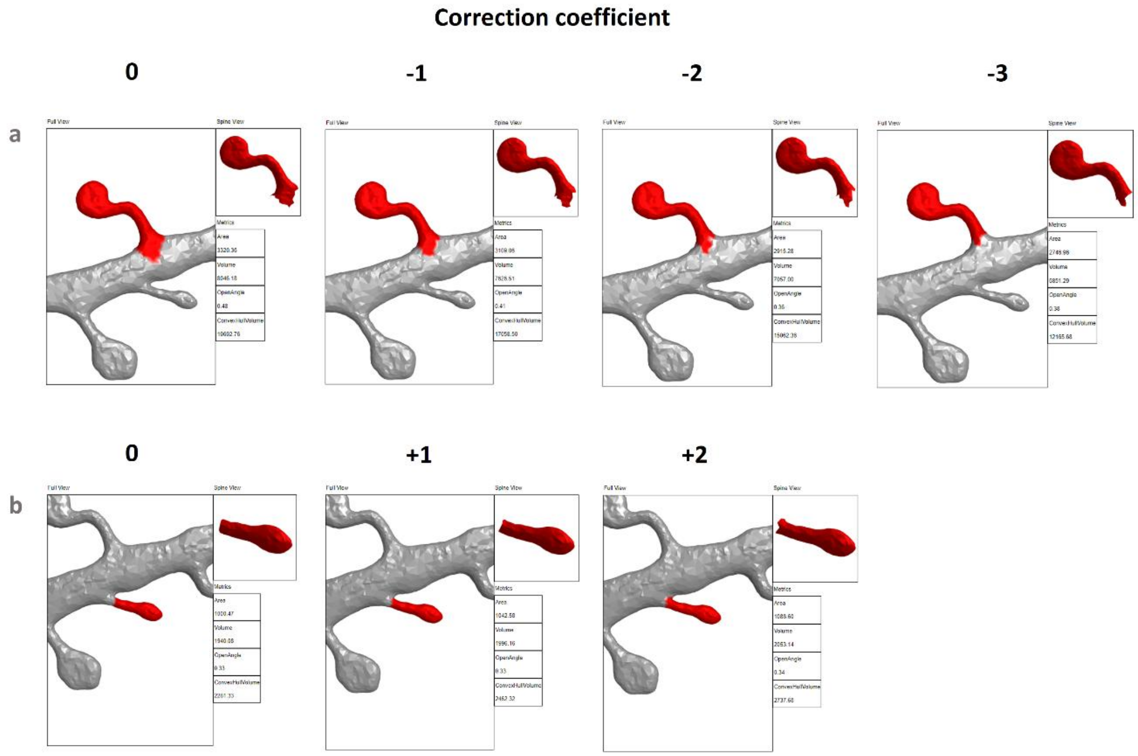

Fig. S1. Manual adjustment of correction coefficient upward (a) and downward (b) in order to achieve better spine basement segmentation.

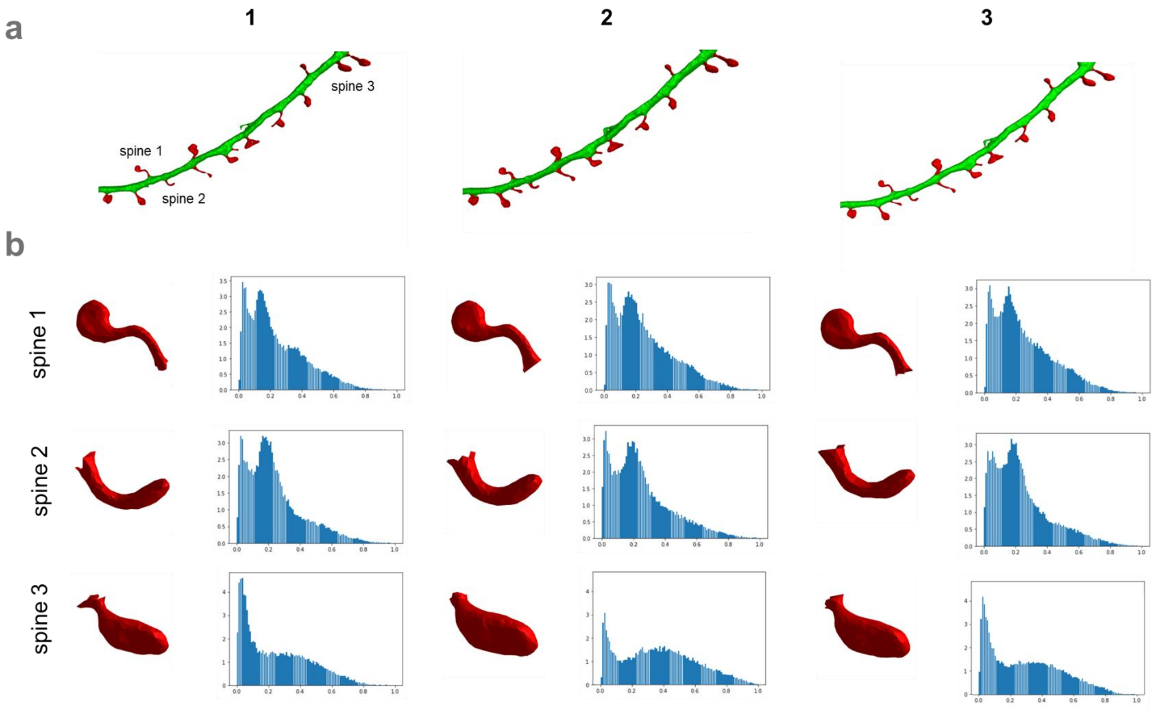

c

	spine 1			spine 2			spine 3		
	1	2	3	1	2	3	1	2	3
Open angle	0,49	0,57	0,54	0,71	0,86	0,75	0,73	0,80	0,80
CVD	0,43	0,39	0,43	0,38	0,32	0,32	0,40	0,38	0,37
Average distance	0,99	1,04	1,01	0,59	0,65	0,66	1,10	1,12	1,07
Length/volume ratio	4,55	2,94	3,65	8,87	5,27	6,08	1,96	1,61	1,65
Length/area ratio	0,38	0,29	0,33	0,44	0,33	0,34	0,25	0,25	0,23
Junction area	0,21	0,46	0,27	0,20	0,36	0,35	0,78	0,37	0,70
Length	1,59	1,66	1,67	0,94	1,01	0,98	1,79	1,82	1,70
Area	3,97	5,26	4,71	1,94	2,75	2,51	6,50	6,94	6,70
Volume	0,35	0,56	0,46	0,11	0,19	0,16	0,91	1,13	1,03
Convex hull volume	0,69	1,04	0,86	0,24	0,37	0,35	1,36	1,28	1,36
Convex hull ratio	0,99	0,85	0,88	1,27	0,91	1,21	0,49	0,14	0,32
Average variation	14,50%			17,30%			12,70%		

Figure S2. Validation of segmentation procedure among experts. a) 3 independent expert's segmentation of the same test dendrite image. b) Enlarged representative images of 3 different spines marked on the panel a) with corresponding CLDH distribution. Notably, the shape of the segmented spines differs in the minor features and the shape and peak position of histograms is conversed. c) The table of numerical morphological features values for showed above representative spines calculated on the expert's segmentation. Average variation for all features lies from 12,7% to 17,3%, where junction area and convex hull ratio are the notably most variable features.

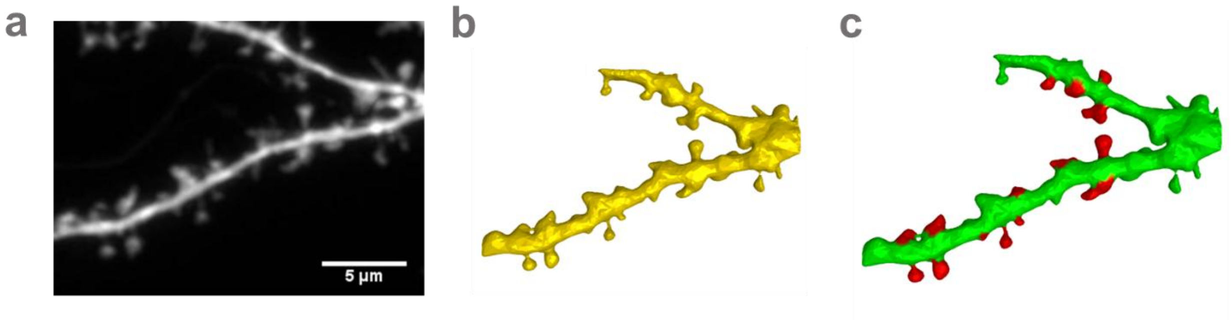

Figure S3. Validation of segmentation procedure on public available dataset. a) Maximum intensity projection from public available 3d .tif confocal dendrite image from with 0.069 x/y and 0.2 z dimensions available at https://sites.google.com/view/3dSpAn/. b) Corresponding built surface mesh. c) Detected with our script protrusions labeled with red color. Example image consists of various overlapping spines, significantly lower resolution and higher noise, nevertheless the subset of spines are detected. Using much higher as possible resolution images is recommended for 3 dimensional reconstruction applications. Development of spines neck reconstruction and spine separation tools should be considered as future direction for the developed software enhancement.

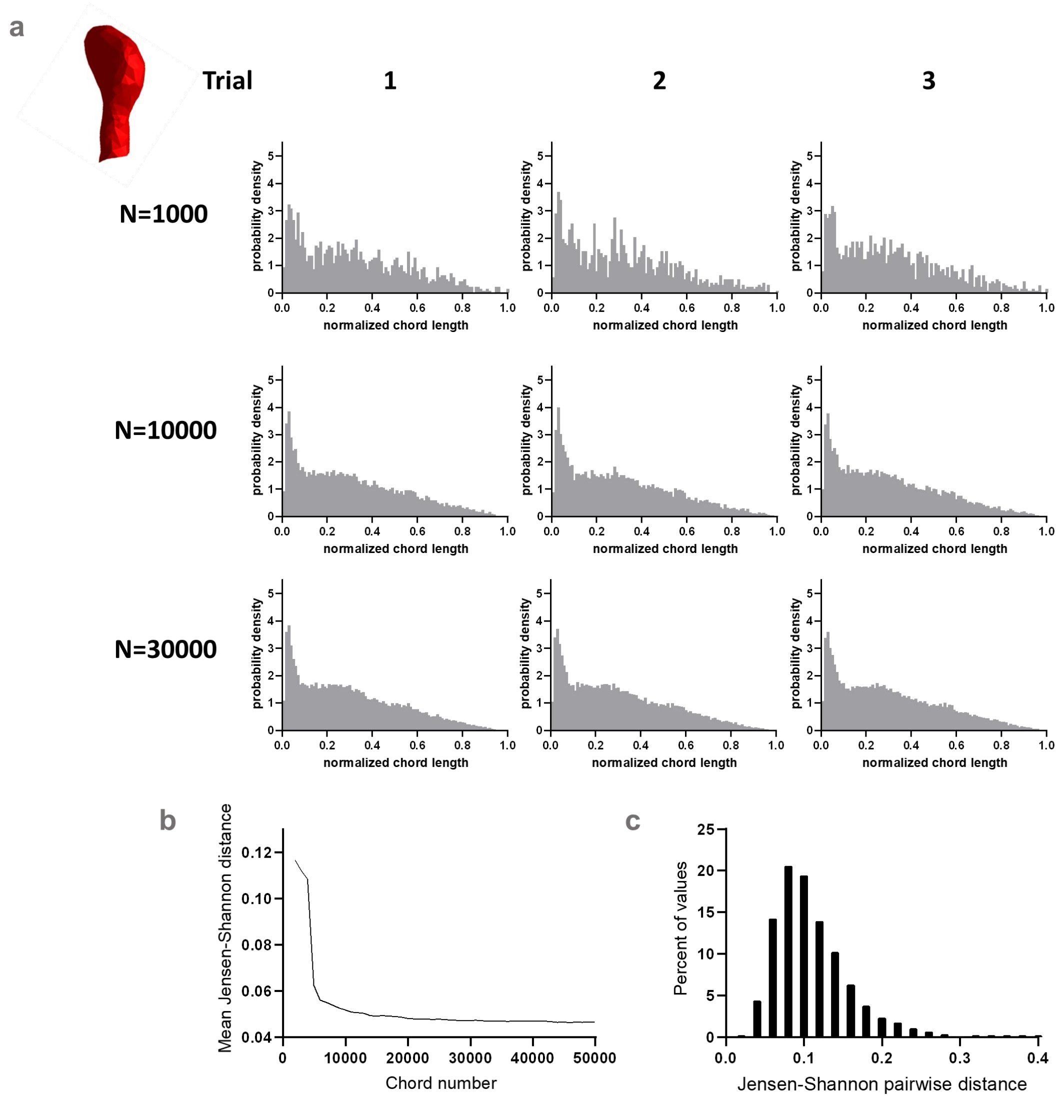

Figure S4. Chord length distribution histograms fluctuations in dependence of chords number (N) and trial. Since the chords are build inside the spine randomly the histogram of their length distribution is depending on their number. a) For small number of chords N=1000 fluctuations on the graph are clearly visible. For N=10000 are only very low amplitude fluctuations are left. N=30000 was defined as an optimal number of chords due to the very low fluctuations left and acceptable computation time. Further increase in chords number do not lead to improvement but requires more computation time. b) Average Jensen-Shannon distance over dataset between CLDH descriptors with adjacent values of number of chords incremented by 1000. After n=30000 the distance reaches the plateau, which is consistent with graph depicted at panel a) and means that future increase in the chords number will not increase the CLDH accuracy. c) Normalized distribution of Jensen-Shannon pairwise distance in dataset characterized with CLDH with n=30000 chord number. Obtained distribution allows to conclude that there are no spines with the same CLDH in our dataset (otherwise the distance will be =0) with the minimum distance is more than 0.036.

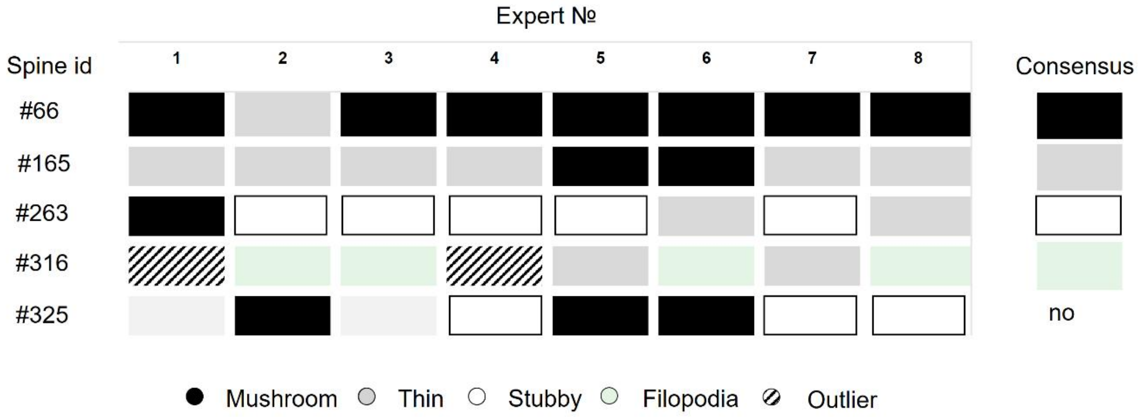

Figure S5. Experts' consensus on spines belongings to the group during manual classification. During assessment of dataset each of the 8 experts labeled spines as *mushroom*, *thin*, *filopodia* or *outlier* (did not fit any of the classes). Consensus was determined when the prevalent number of votes existed, otherwise spine was labeled as non-consensus and it was excluded from training and test datasets.

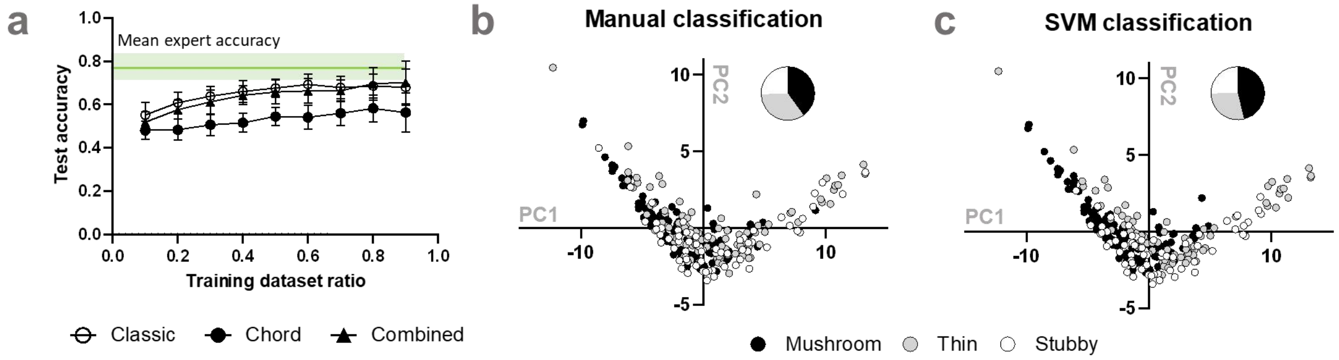

Figure S6. SVM-based dendritic spines classification with Laplasian Radial Basis Function kernel. a) Accuracy of SVM classification with Laplasian Radial Basis Function kernel as a function of training dataset ratio for classic (solid circles), CLDH metrics (black circles) and their combination (black triangles) for n=30 trials. Data shown as mean±SD. b, c) Spines classification map with classic metrics in two-dimensional principal components coordinates based on manual (b) and SVM (c) classification. For panels b and c mushroom spines are shown in black, thin in gray and stubby in white.

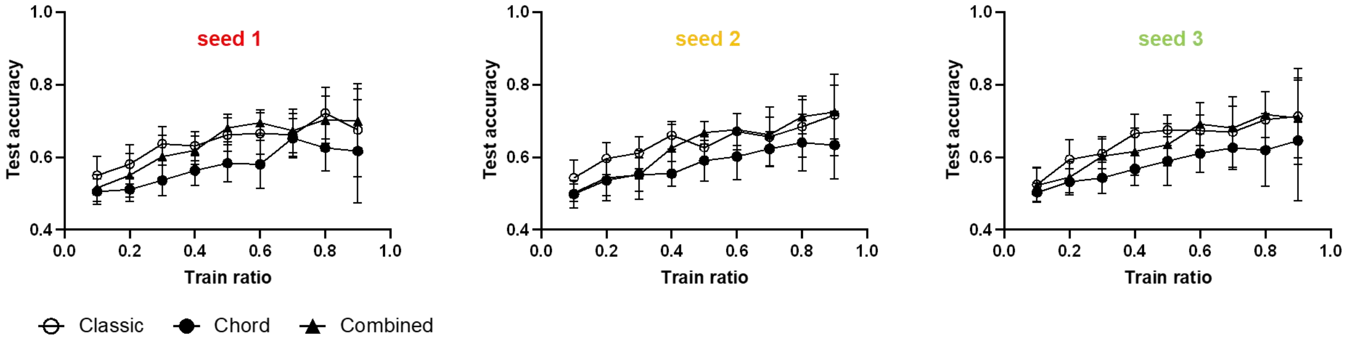

Figure S7. SVM-based dendritic spines classification with Laplasian Radial Basis Function kernel with various seed. Example of averaged test accuracy of SVM classification over n=20 runs as a function of training dataset ratio for classic (solid circles), CLDH metrics (black circles) and their combination (black triangles), with manually selected seed for training. Data shown as mean±SD. Notably, combination of metrics from particular training dataset ratio outperform or equal to classics metrics, but it does not reach statistical significance.

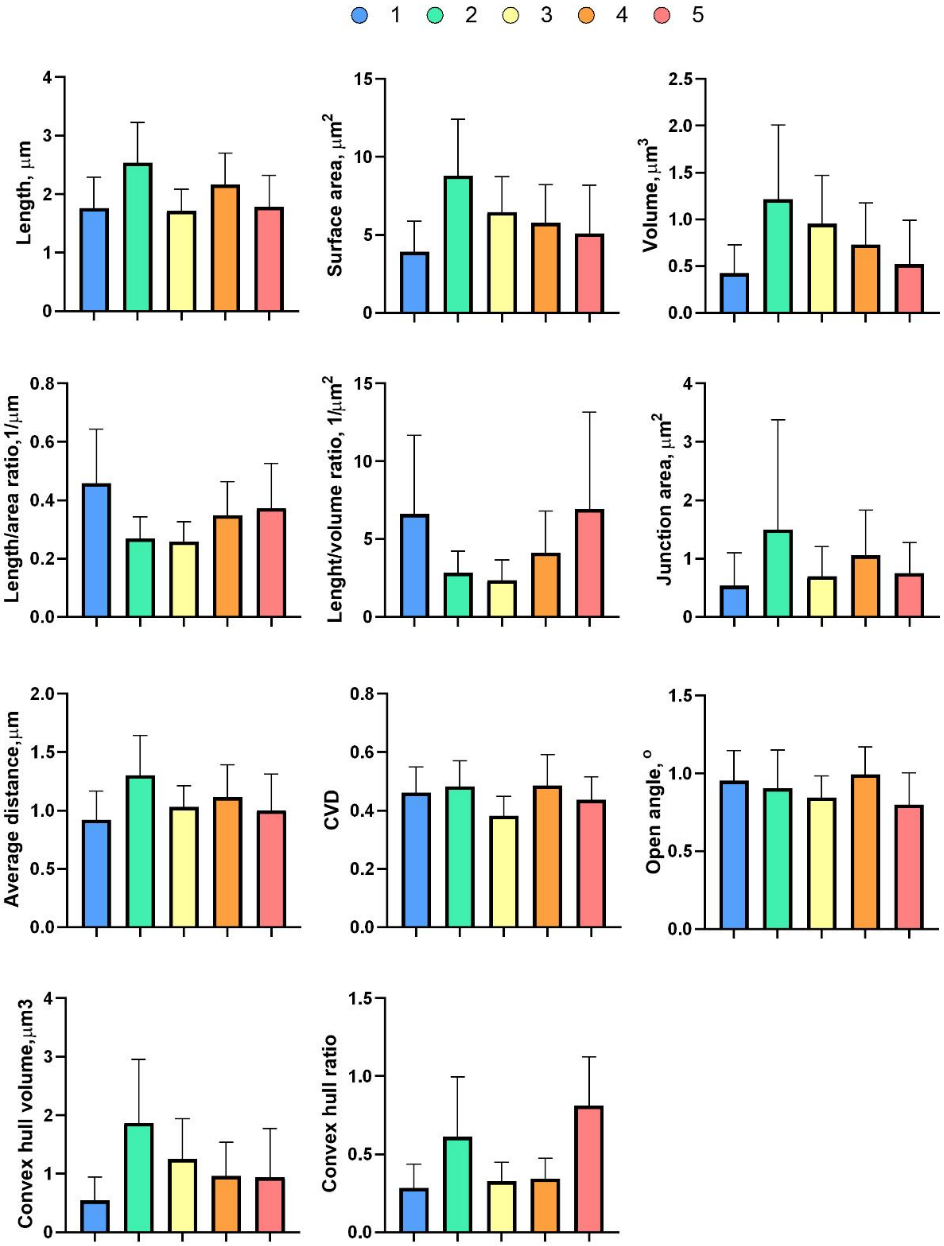

Fig. S8. Distribution of geometrical metrics among the 5 clusters defined by k-means clustering using chord metric with maximum class variation score.

Eleven metrics (labeled on Y axis) - spine length, surface area, volume, length/area, length/volume ratio, junction area, average distance, coefficient of variation in distance (CVD), open angle, convex hull volume, convex hull ratio are shown for each cluster (color coded fo clusters 1 – 5) as mean±SD. Description of metrics are provided in Materials and methods section. For all metrics medians are not equal according to Kruskal-Wallis test ($p<0.0001$).

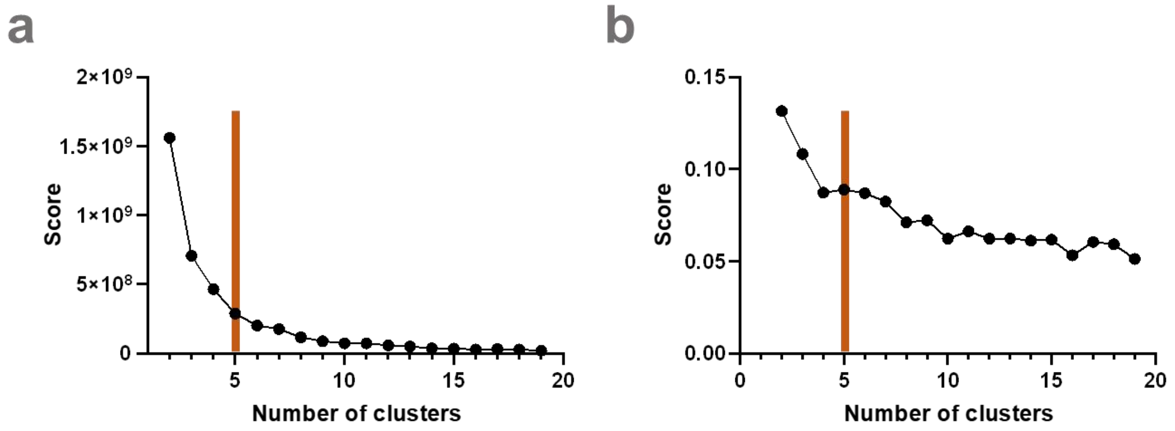

Fig. S9. Determination of cluster number in k-means clustering in CLDH feature space using elbow and silhouette method.

Clustering coefficient score in dependence on cluster number for a) elbow and b) silhouette methods. The optimal according to used criteria number of clusters n=5 is labeled with line. Corresponding clustering map in two-dimensional principal components coordinate, where clusters are labeled with different colors, is shown on Fig.4.

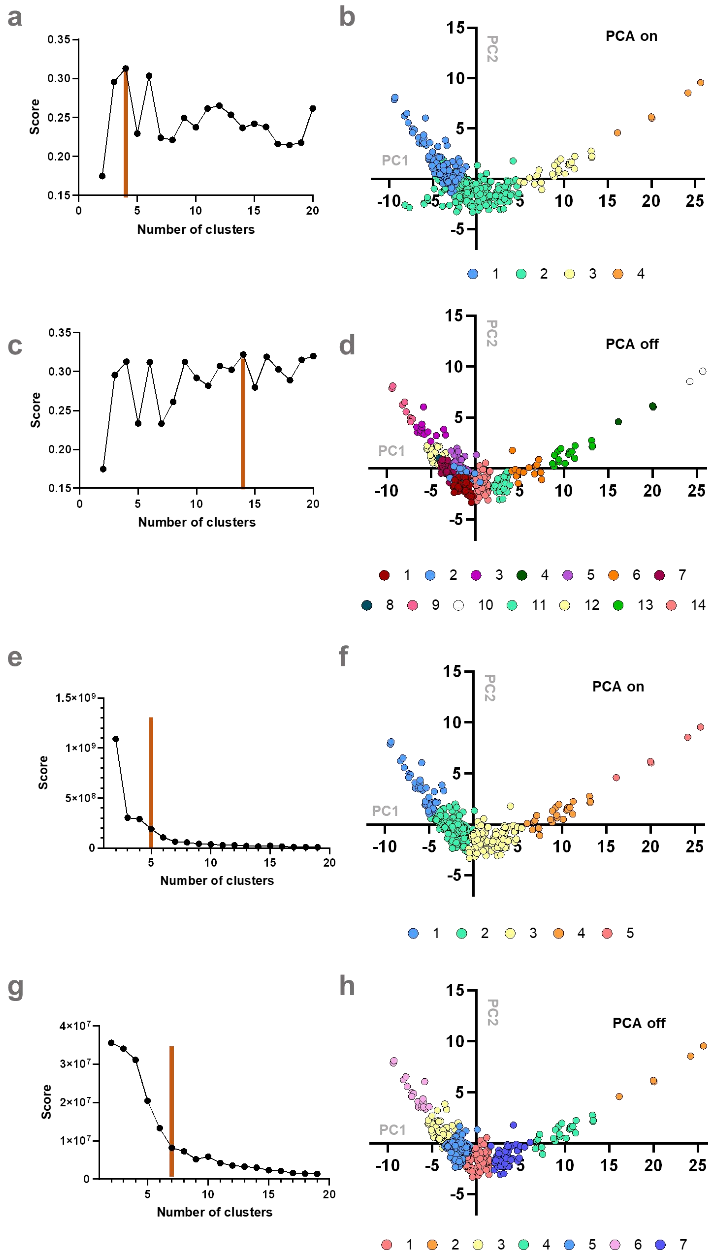

Fig. S10. Dendritic spines k-means clustering in classic feature space using max class divergence criteria and elbow method.

Max class divergence score in dependence on cluster number for classic feature space a) with PCA and c) no PCA. The point of maximum value on the curve n=4 and n=14 is labeled with line. b, d) Corresponding clustering map in two-dimensional principal components coordinate, where clusters are labeled with different colors, with PCA and no PCA. Distortion score in dependence on cluster number for classic feature space e) with PCA and g) no PCA. The point of inflection on the curve n=5 and n=7 is labeled with line. d, f) Corresponding clustering map in two-dimensional principal components coordinate, where clusters are labeled with different colors, with PCA and no PCA.
