# Supplementary material for: SpineTool is an open-source software for analysis of morphology of dendritic spines
Source: Sci Rep. 2023 Jun 29;13:10561. doi: 10.1038/s41598-023-37406-4 (PMC10310755; doi:10.1038/s41598-023-37406-4)
Supplement: Supplementary file 2 — Supplementary Information. [file 41598_2023_37406_MOESM2_ESM.pdf]

Supplementary 2 for “SpineTool is an open source software for analysis of dendritic spines morphology”

SpineTool Tutorial.

1. Installation

System requirements:

- Windows 8 or newer
- Minimum 1GB RAM
- Minimum 6 GB disk space

Installation steps:

1. Download and unzip the code folder from https://github.com/spbstu-applied-math/SpineTool
2. Unzip CGAL files next to code, e.g. `PATH_TO_CODE\CGAL\...` and put example_dataset or other dataset files next to code, e.g. `PATH_TO_CODE\example_dendrite\`... or `PATH_TO_CODE\example_dendrite.tif`.

   Note that PATH_TO_CODE must consist of directories containing only Latin letters in order to be able to use a jupyter notebook in this directory.
3. Install Anaconda and open Anaconda Promt
4. Execute:

```
cd PATH_TO_CODE
conda create --name spine-analysis -c conda-forge --file
requirements.txt –y
```

5. To run the software open Anaconda Promt, execute:

```
cd PATH_TO_CODE
conda activate spine-analysis
jupyter notebook
```

The joined Jupiter Notebook, containing separated notebooks, will open:

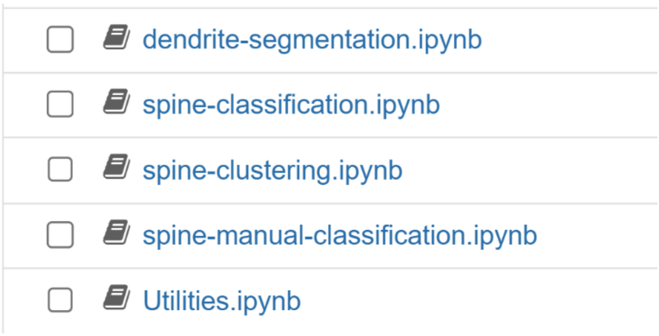

Click on required notebook to start work. Notebook descriptions are provided below.

2. Example dataset

With the source code, we include an example dataset with expert partitioning named “0.025 0.025 0.1 dataset”. This dataset consists of 275 polygonal meshes of dendrite spines related to 54 dendrites and of 54 polygonal meshes for dendrites. A dataset subdirectory named "manual_classification" contains the expert markups from 8 people obtained using the developed software and the results of merging the classifications to obtain a consensus classification.

Also, dataset named “example_dendrite” is included with the source code to use in segmentation and utilities notebooks. It consists of a .tif image of a dendrite, 22 polygonal meshes of dendrite spines and a dendrite polygonal mesh.

3. Dendrite Segmentation Notebook

The dendrite segmentation notebook is used to extract a dendrite polygonal mesh from tiff images of z layers of the dendrite photo and to select polygonal spike meshes from the dendrite mesh. This code produces files in format .off with dendrite mesh (surface_mesh.off) and spines meshes (spine_{i}.off) extracted from the image.

3.1. Uploading

At first step user need to specify image resolution at X-Y and Z-axis.

Image Binarization

Set image sampling density in micrometer/pixel.

```
В [ ]: sampling_x = 0.025
       sampling_y = 0.025
       sampling_z = 0.1
```

Then to open image and perform segmentation is necessary to specify path to the image. Image is required to be 8-bit depth multipage tiff. Image containing folder need to be placed at the PATH_TO_CODE folder. The dataset path to the dendritic .tif images must be specified in the second block of the notebook code.

Note that preliminary images need to be cropped to contain one meaningful dendrite. Please avoid dendrite intersection since it may interfere with segmentation results.

set dataset path

```
import numpy as np
from spine_segmentation import load_tif
from notebook_widgets import show_3d_image
from scipy.ndimage import gaussian_filter
import os

image_path = "example_dendrite.tif"
image_name = os.path.basename(image_path)

# Load tif
image: np.ndarray = load_tif(image_path)
image = gaussian_filter(image, 1)
show_3d_image(image)
```

3.2. Image inspection

After image uploading by suing x/y/z slider you may visualize your image at X-Y and Y-Z planes.

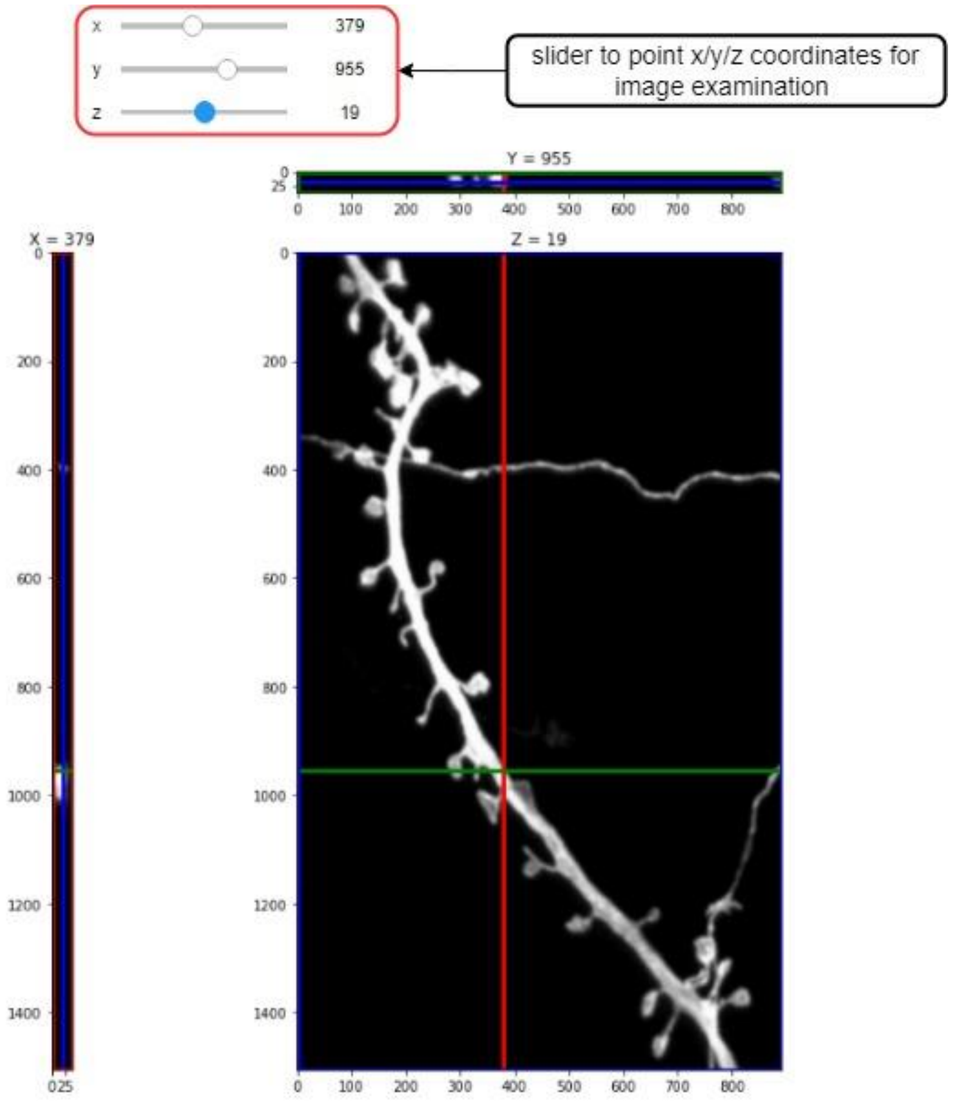

2.3 Binarization

Image is segmented according to adaptive binarization algorithm where threshold is calculated locally in dependence on nearest pixels intensity. Base threshold, weight and block size parameters are adjusted the better binarization.

We recommend using high weight parameters in the 90-100 range and low block parameters in 1-3 range. The binarization result is visualized by green mask, covering the gray image. The mask opacity might be adjusted in dependence of user preferences.

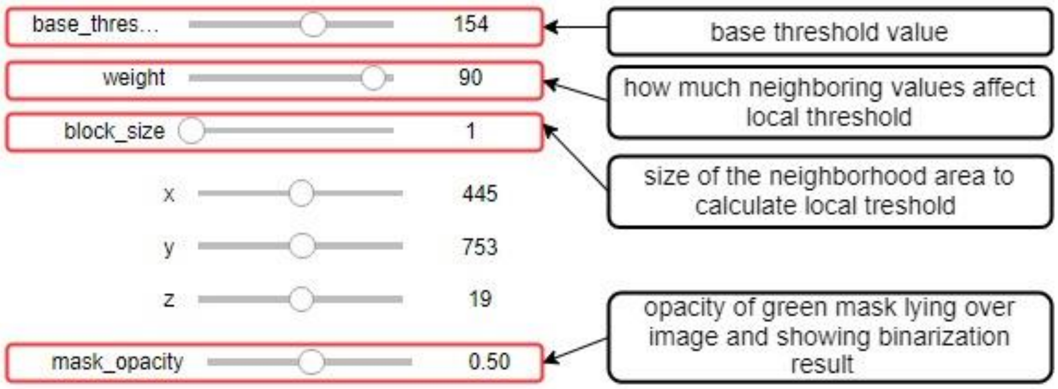

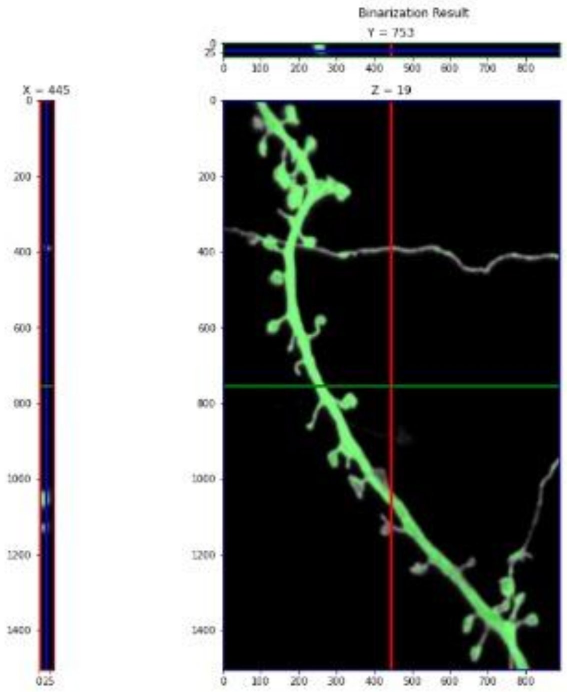

2.4 Connected component selection

Connected component – is the segmented area presenting single object. After binarization several connected components are generated in dependence of the image. The biggest one is selected a priory (colored in white) and usually manual selection is not required. You may control the dethatching spines appearance - they will be colored in dark gray, and if necessary, go back one step to segmentation and try other parameters.

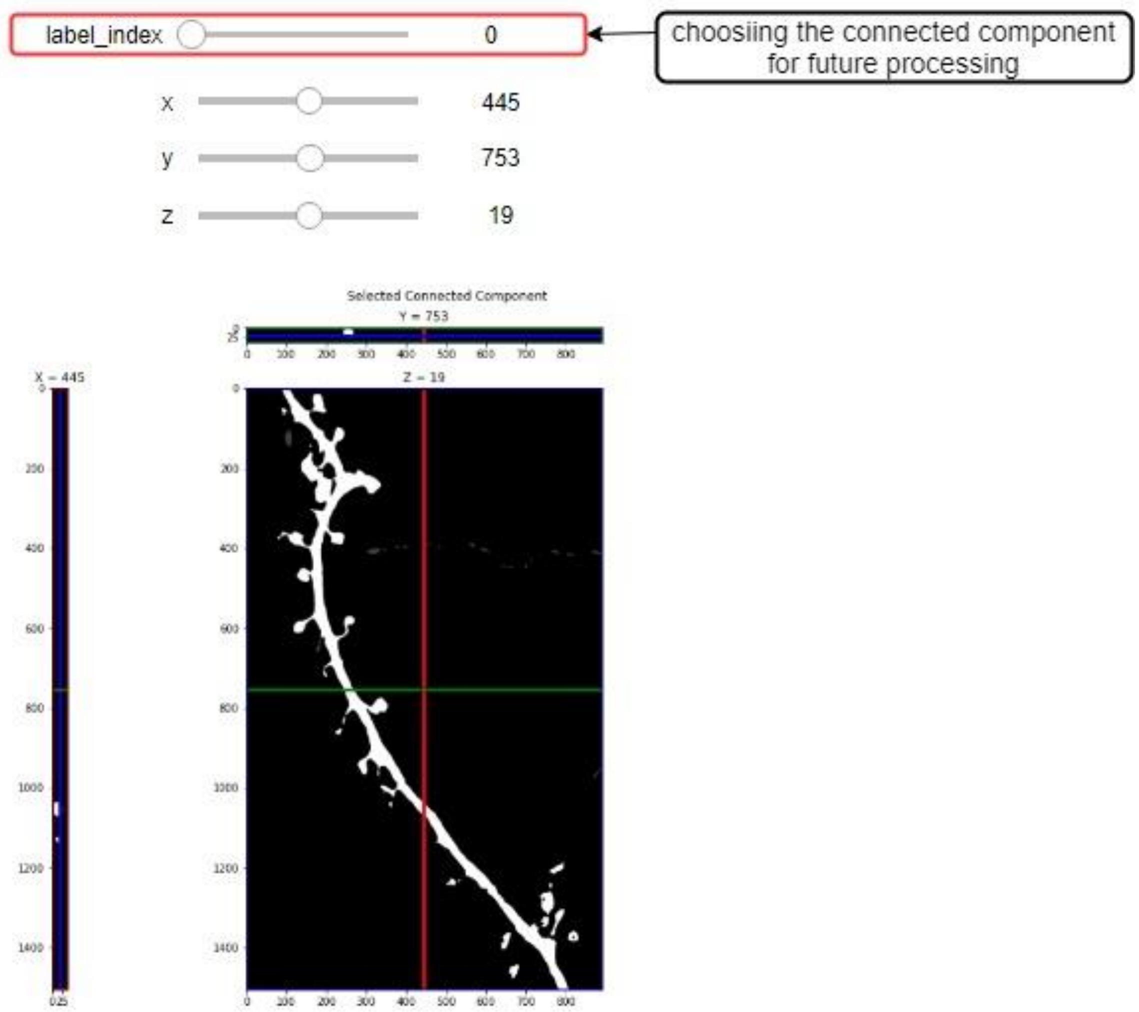

2.5 3D surface construction

At this part, 3D mesh surface is generated basing on the segmented image. It is time-consuming process, which depends on image size. As default z-display factor is 0.5.

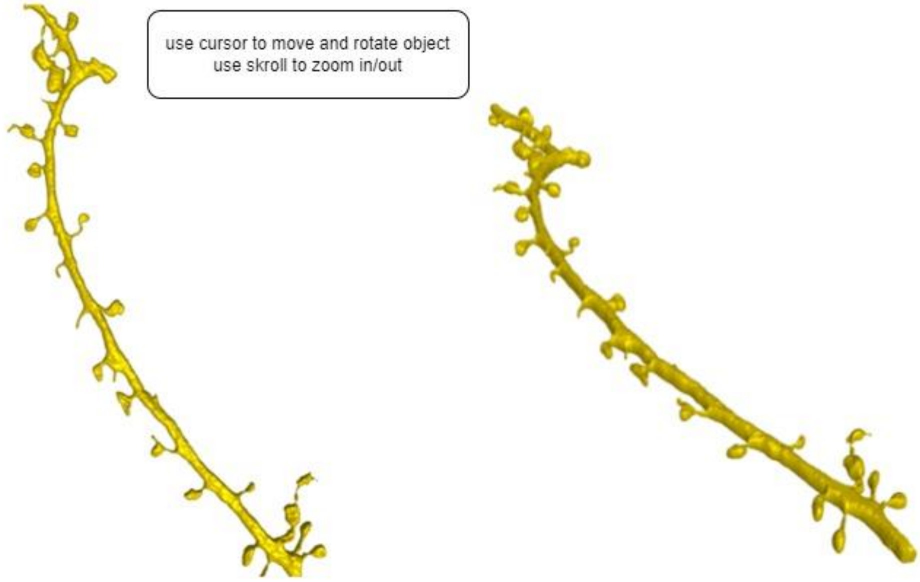

2.6 Segmentation

At the next step spines are segmented from the dendrite. Sensitivity — how much distance from skeleton affects segmentation. Higher values will result in less false positive spines, but worse segmentation at spine base and detection of smaller spines. Correction parameter

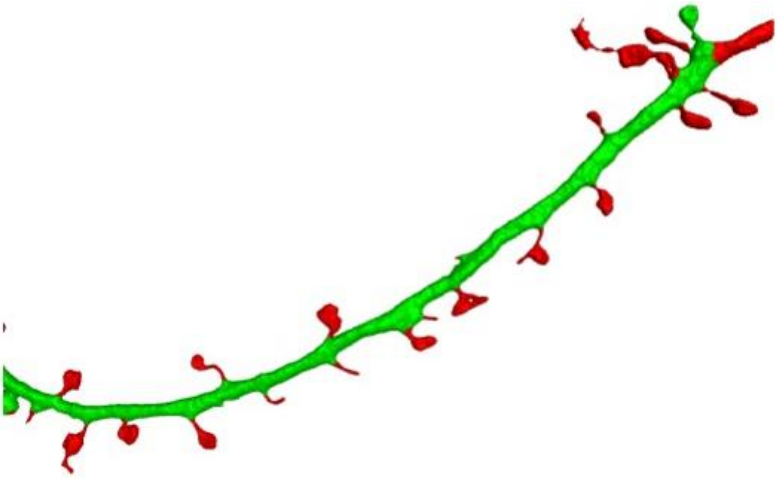

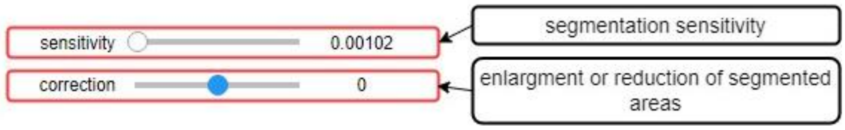

2.7 Spine's segmentation correction

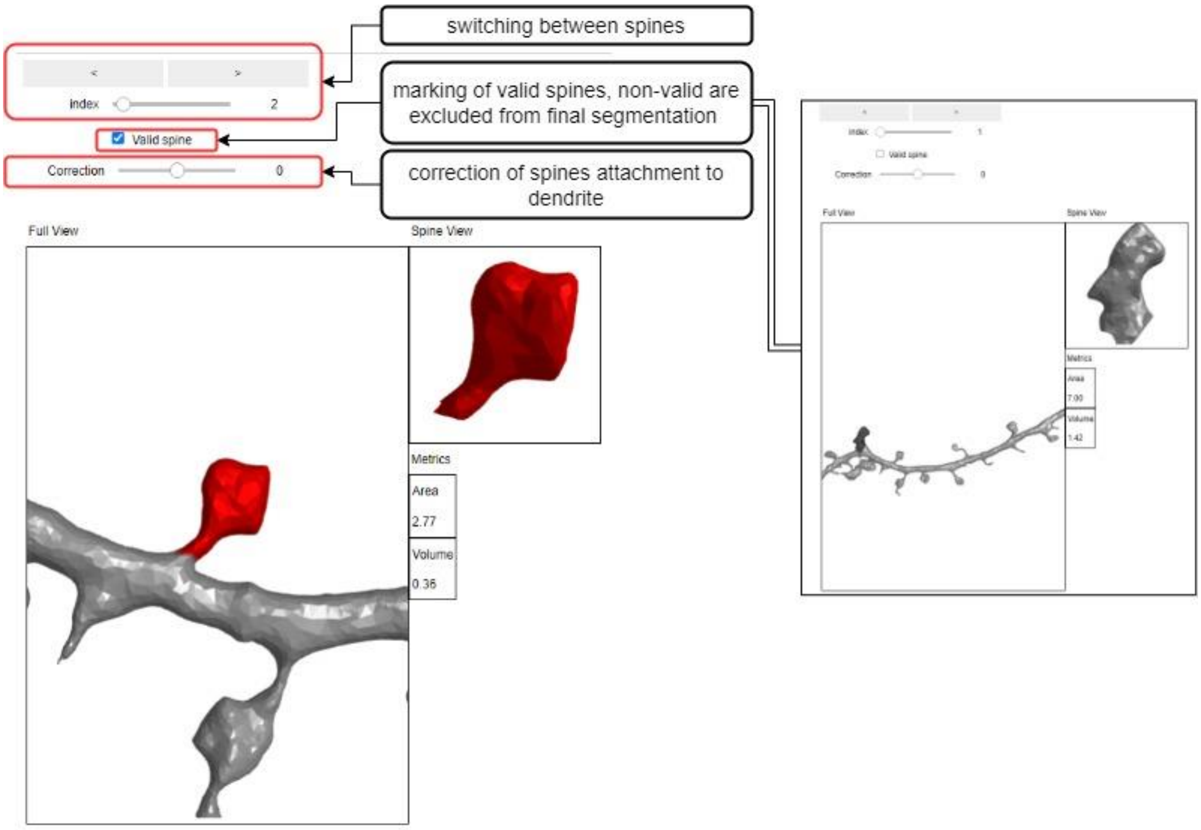

2.8 Final segmentation

At the last part final segmentation is generated from manually filtered spines. Spine meshes are saved to *"output/spine_{i}.off"* files.

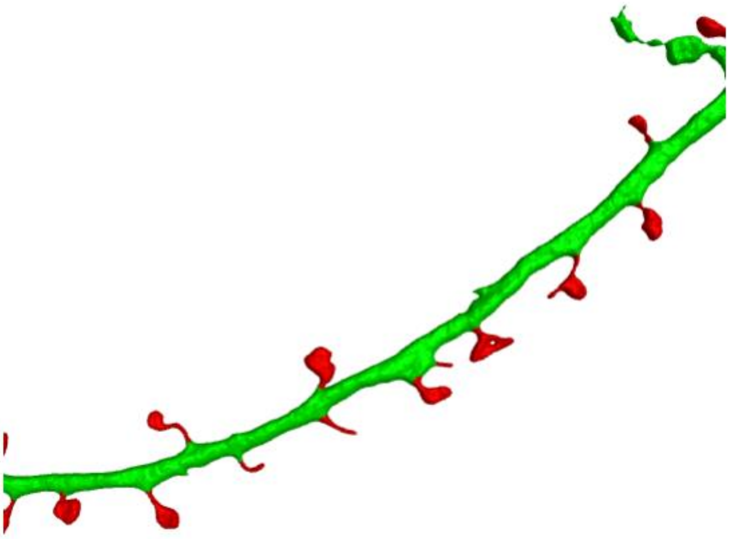

3 Manual Classification Notebook

This notebook is used to create an expert classification or view an existing spine grouping by classes. After notebook execution, the user receives a .json file with spines classification and can review the spines and the class assigned to them by switching within the widget between the representation of spine characteristics.

3.1 Required input and preparations

To perform manual classification, you should have a data set of spines and their dendrite polygonal meshes obtained by executing code from the Dendrite Segmentation Notebook. It is also necessary to have metrics for these spine meshes, which users can produce by running the code from the "Calculate Metrics" section of Utilities Notebook.

Firstly, a user should have segmented spines and dendritic meshes stored in .off files and obtained after executing commands from the «Dendritic segmentation» notebook. Secondly, the path to the spines and dendritic meshes dataset must be specified in the first block of code of the «Utility» notebook.

Please note that according to the Getting Started section your dataset should be placed next to the code, e.g. `PATH_TO_CODE\example_dendrite\`... Otherwise you need to specify the absolute path to the dataset. Also make sure that the string value of the path is correct: separation symbols "\" are preceded by the escape character.

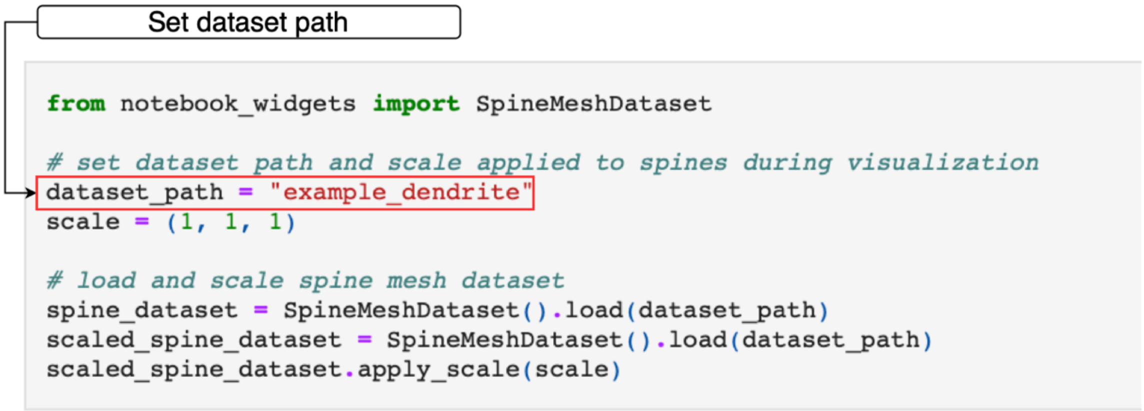

After executing this first block of code, the user must run the "Calculate Metrics" section, in which the classical metrics and chord metric are evaluated and the results are saved to a .csv file to the dataset folder for further use.

The path to the prepared dataset must be specified in the first block of the Manual Classification Notebook code in the dataset_path variable. The user needs to specify the path to the file where the manual classification will be stored and the list of class names in its classification. In the case where the variable classification_save_path specifies the path to an existing spine grouping file, the list of class names should match the classes saved in the file.

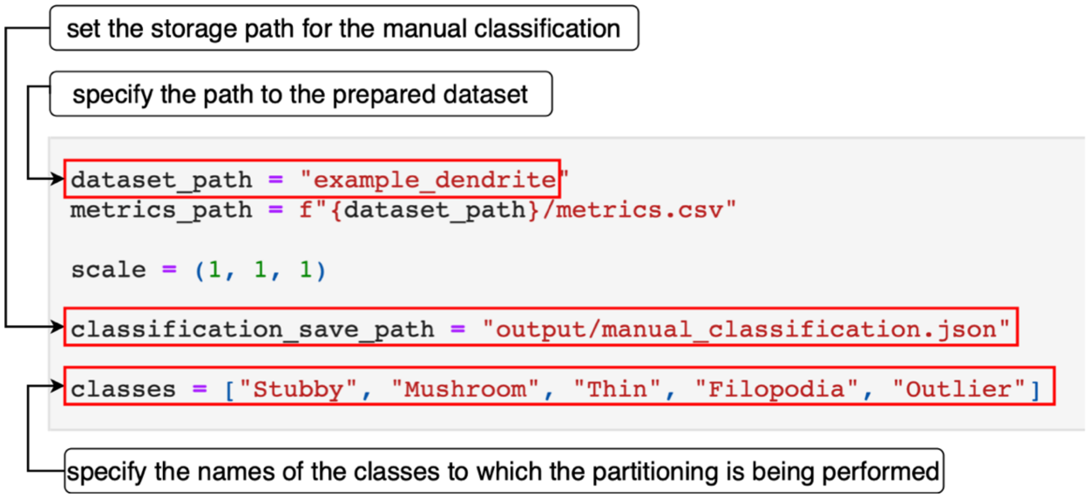

3.2 Classification widget

The manual classification widget allows to examine spine features and assign its class by clicking on the button with the respective name. After selecting a class and pressing the corresponding button, the spine in the widget automatically switches to the next one, the position of the spine_index slider changes.

To switch between the spines for classification revision use this slider and buttons. The color of the classified spike will match the color of the button whose name matches the class of the spike.

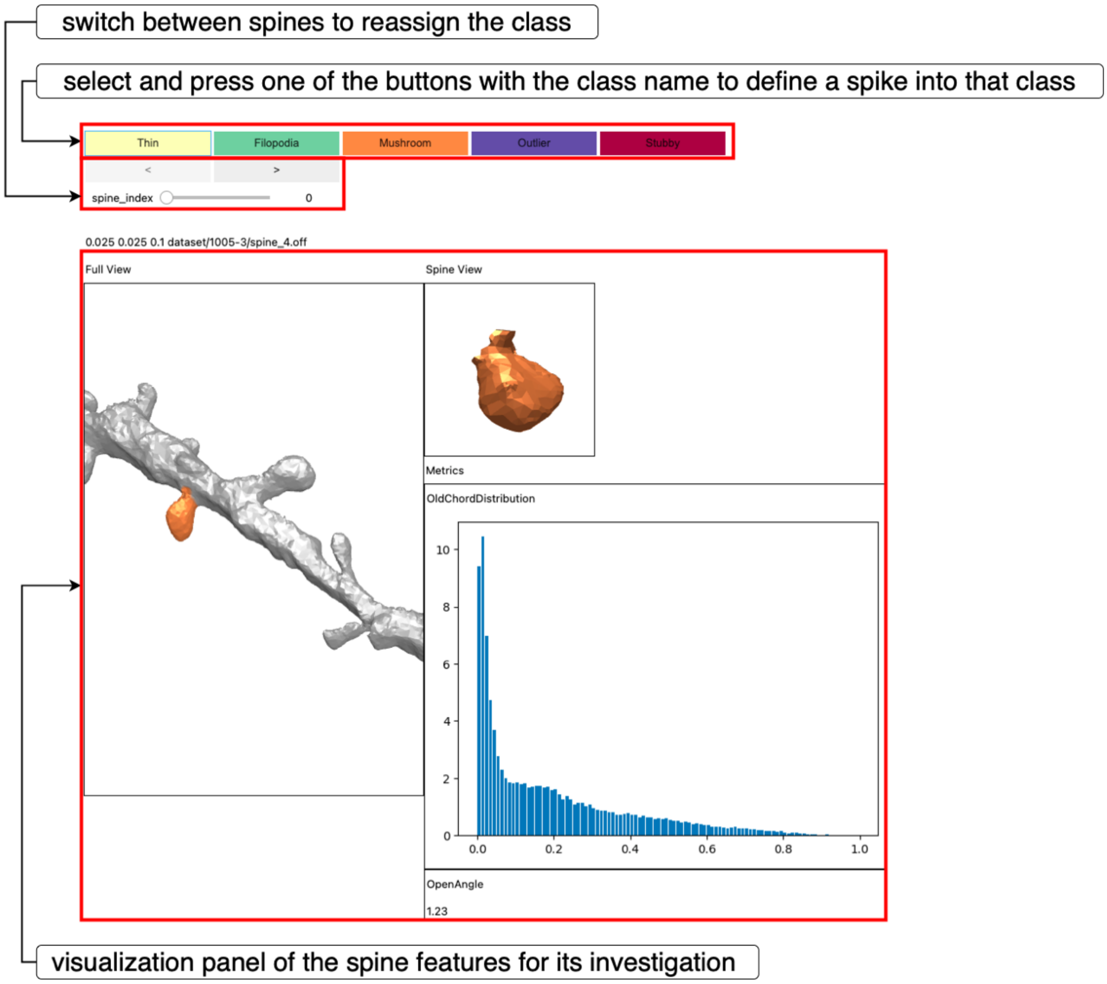

3.3 Export results and output structure

To save the classification the user should execute the last block of Manual Classification Notebook code where the classification is extracted from the widget, the classes are visualized in the coordinates of the principal components of the metrics and the grouping is saved.

Classification is saved in .json format of the same structure as the clustering in Spine Clustering Notebook: the file contains three sections - "groups", "samples" and "outliers_label". Here "outliers_label" is set to "Unclassified" in case the classification is created from scratch and not loaded from an existing file.

4 Spine Classification Notebook

Notebook is used to train a model to classify spines by classical metrics and chord metrics based on manual labeling into 3 classes: mushroom, thin and stubby. The support vector machine is taken as a model for training. After training, the model with the best accuracy is selected for each of the configurations of the set of metrics used: classical metrics, chord metric, and a combination of classical metrics and chord metric. This model is used to classify the entire dataset. This model is used to classify the entire dataset. The resulting classification is compared to the manual reference classifications and saved to a .json file.

4.1 Required input and preparations.

To train the classification model, the user should prepare a dataset with polygonal meshes of spines and dendrites, obtained by executing code from the Dendrite Segmentation Notebook. It is also necessary to have in the dataset the metrics for these spine meshes, computed using the Utilities notebook. These steps are similar to those described in the data preparation section of the Spine Clustering Notebook. Also the training of the model requires a reference classification to be trained on. The reference classification can be obtained using the Manual Classification Notebook.

The path to the prepared dataset must be specified in the first block of the Spine Classification Notebook code in the dataset_path variable. User also could specify the metrics reduction method that would be used for visualization. Two methods are supported: pca and tsne:

```
dataset_path = "0.025 0.025 0.1 dataset"
show_method = "tsne"
# show_method = "pca"
```

In the second block of the Spine Classification Notebook code user should specify the path to the reference classification for model training.

set the storage path for the manual classification

```
from spine_metrics import SpineMetricDataset
from spine_fitter import SpineGrouping

merged_grouping = SpineGrouping().load(f"{dataset_path}/manual_classification/manual_classification_merged_reduced.json")

metrics = SpineMetricDataset().load(f"{dataset_path}/metrics.csv")
metrics = metrics.get_spines_subset(merged_grouping.samples)

classic = metrics.get_metrics_subset(["OpenAngle", "CVD", "AverageDistance",
                                      "LengthVolumeRatio", "LengthAreaRatio", "JunctionArea",
                                      "Length", "Area", "Volume", "ConvexHullVolume", "ConvexHullRatio"])
```

4.2 Model training

The "SVM Classification" and "2 Datasets" sections are used to run training of the LinearRegression model from the sklearn package, to tune the model using grid search, to evaluate classification quality, and to perform classification on the entire dataset.

The accuracy of the resulting model is measured depending on the training sample rate for each of the spike metrics configurations. According to the results of training and evaluation of the model, a graph of the accuracy of the classifiers and plots of the resulting classes in the coordinates of the principal components are displayed to the user.

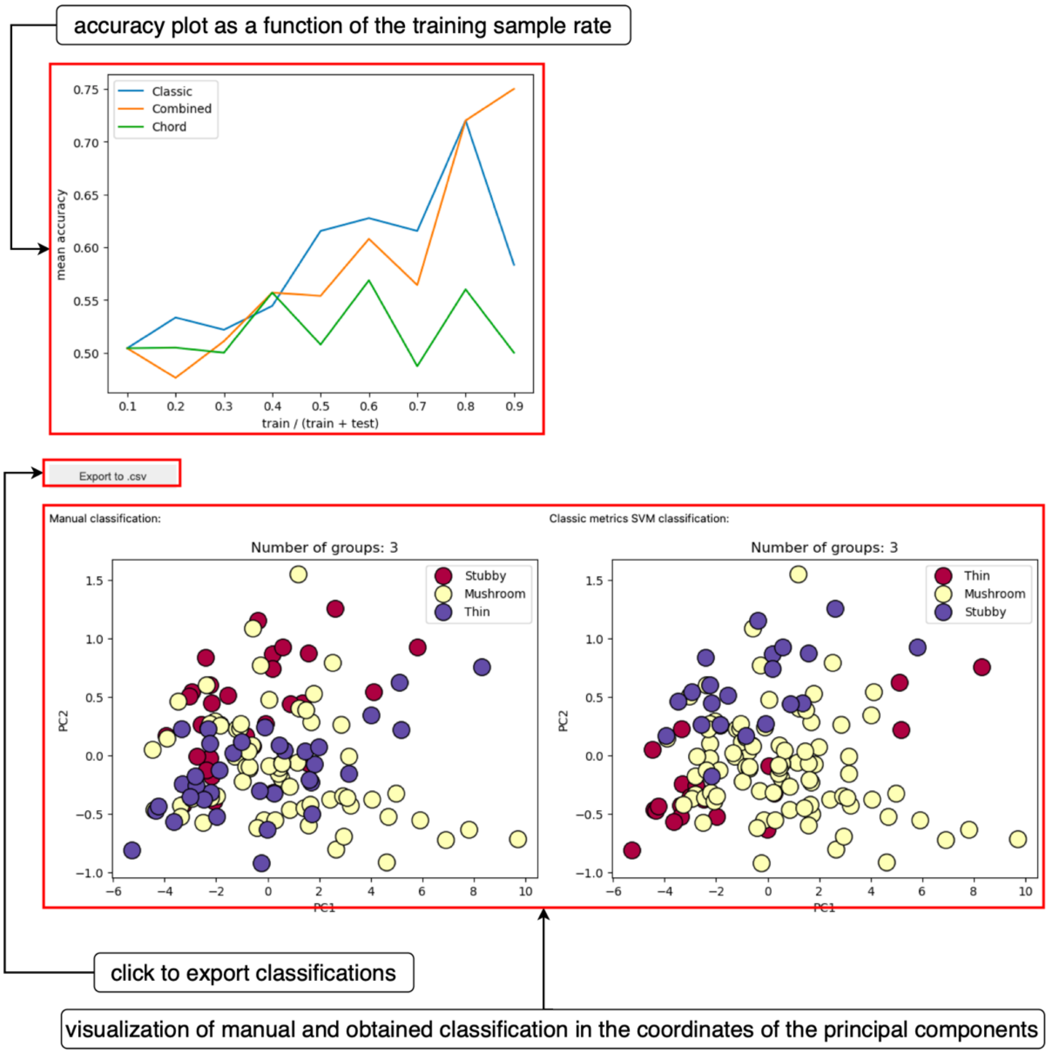

4.3 Export results and output structure

To save the classification the user should push the "Export to csv" button. Classification is saved in .json format of the same structure as the clustering in Spine Clustering Notebook: the file contains three sections - "groups", "samples" and "outliers_label". Here "outliers_label" is set to None. Besides the classification, accuracy graphs for each metrics configuration are saved to the .csv file.

5 Spine Clustering Notebook

Clustering based on classical and chord metrics could be produced by spine clustering notebook. Results are shown in notebook widget in which users can choose clustering by its parameters, see metrics distribution visualization, clusters visualization, iterate through spines' meshes and extract results.

5.1 Required input and preparations

In the first block of code of the «Spine clustering» notebook specify path to the dataset with meshes and calculated metrics. The user can have a manual spine classification produced using a «Spine manual classification» notebook. In case the user has several manual classifications by different specialists, a merged version of classification be used to calculate the clustering score. The merged classification can be obtained by executing the Merge Manual Classifications code block of the «Utilities» notebook. In the case the manual classification was not performed, you can comment out the lines of code where it is loaded and set variable manual_classification to None. User may choose the way to visualize clustering results in two dimensional coordinates Principal component analysis (PCA) or t-distributed Stochastic Neighbor Embedding (tSNE) by tapping «pca» or «tsne» at show_reduction_method parameter correspondingly.

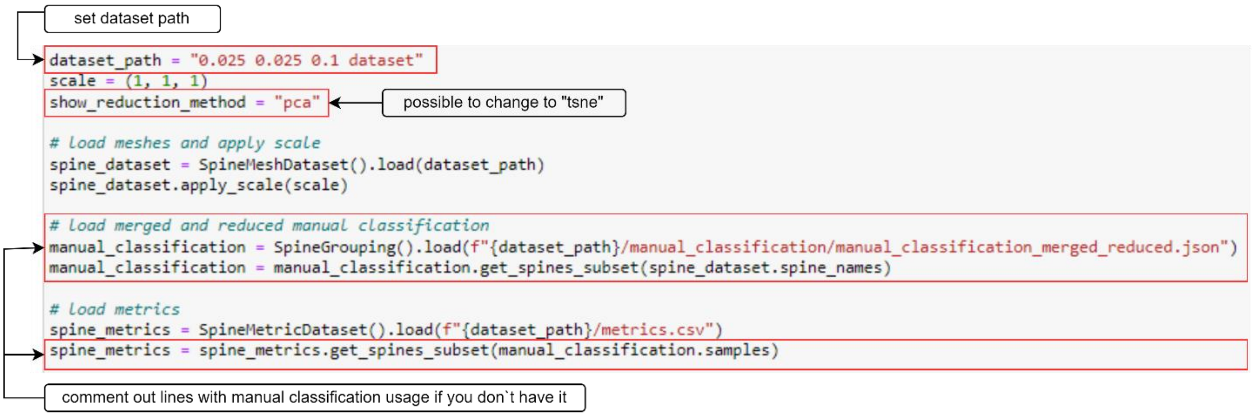

5.2 Clustering methods

The spine clustering notebook has sections called according to the clustering methods and metrics used in them. The k-means and DBSCAN algorithms are used for classical metrics and chord metric on Euclidean distance and Jensen Shannon distance. For all clustering results the elbow score could be used to evaluate the results, implementations are added to the first block of notebook code: kmeans_elbow_score and dbscan_elbow_score functions. For classical and combined metric spaces a silhouette metric can be used to evaluate the results. Its implementation is also added to the first block of code.

To start clustering and draw the widget, it is necessary to call the function corresponding to the selected method: k_means_clustering_experiment_widget for k-means algorithm and dbscan_clustering_experiment_widget for DBSCAN algorithm. Each widget has parameters: spine metrics values, loaded polygonal meshes dataset, callback for computing the clustering index, clustering parameter range - minimum value, maximum value and step; distance to use during clustering, loaded manual classification to compare with the clustering, path for results export. User choose the number of clusters selection method – elbow or silhouette score, or

max max class divergence criteria (lambda clusterizer), firstly proposed and described at the main paper text, by commenting out others with #. At parameter «dim_reduction» user may choose the PCA/tSNE to perform clustering after dimensionality reduction or leave the field empty to perform clustering without dimensionality reduction.

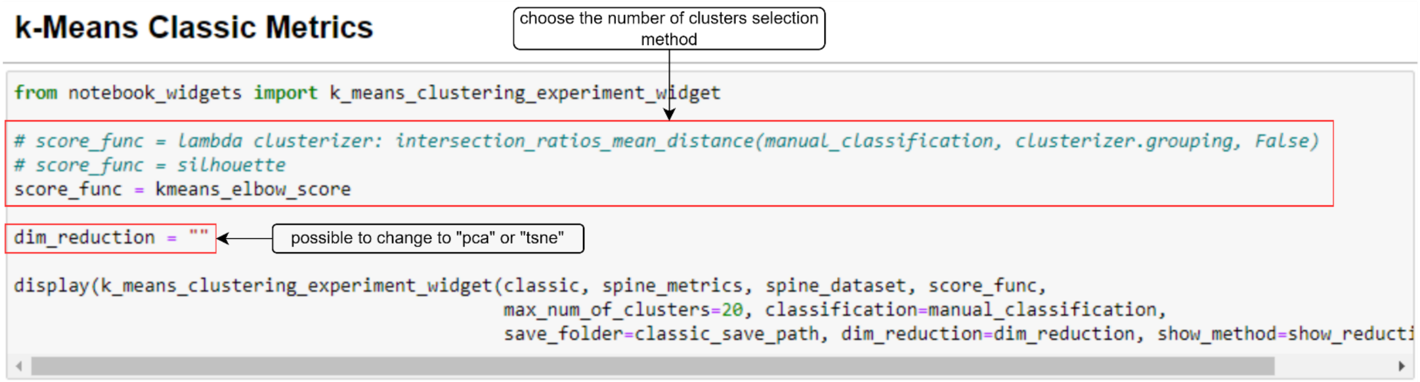

5.3 Clustering Results Widget

Widget has four sections: manipulators to switch between clustering results for different parameters, visualization of the clustering and the elbow/max interclass variation score score for it, a button for exporting the results of the selected clustering, a drop-down list of widgets for evaluation and inspection of the obtained clustering.

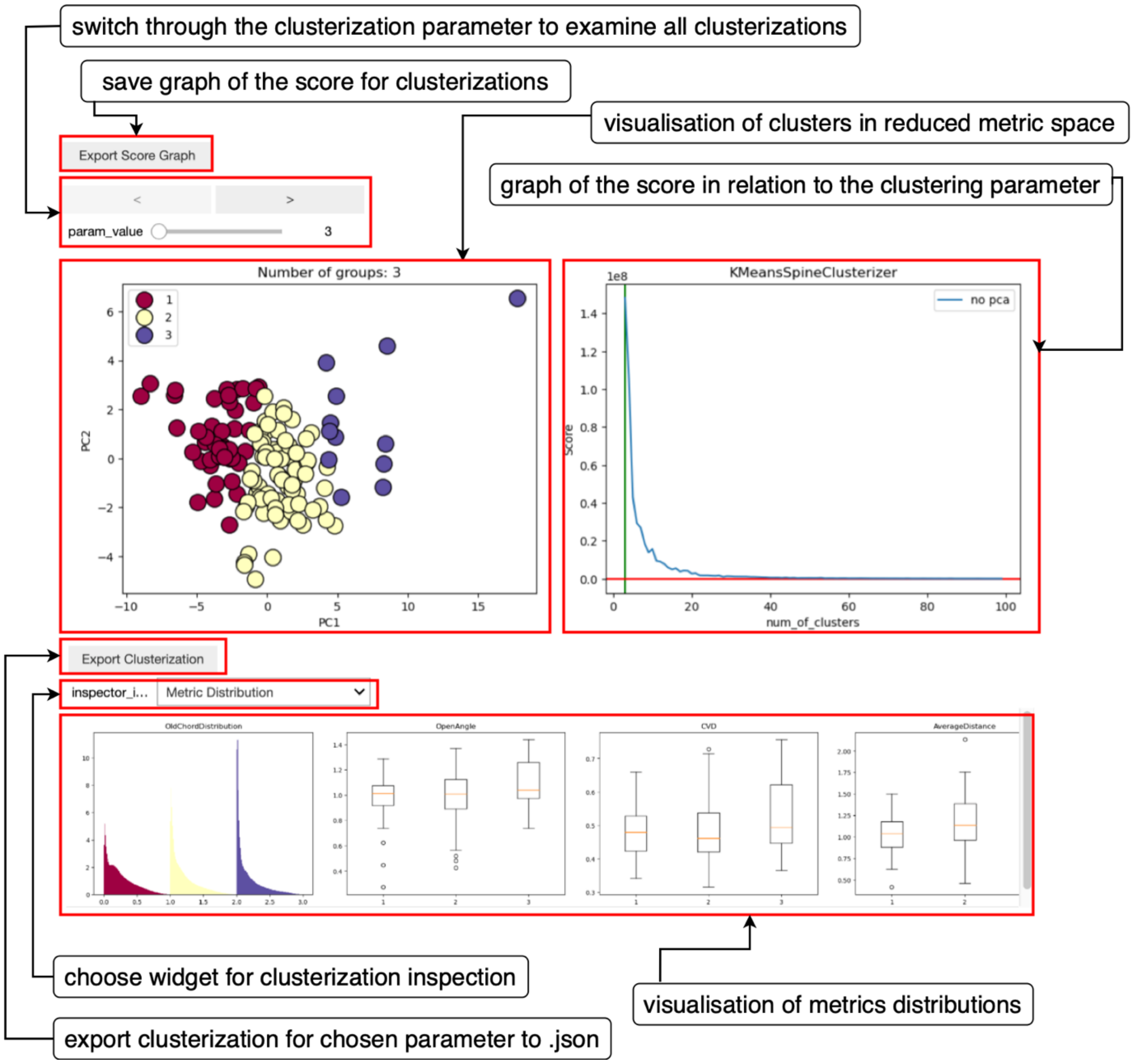

5.3.1 Visualization of clusters

The visualization is obtained by principal component analysis on the computed metrics. Also next to the plot of the spine positions in the principal component coordinates the graph of the clustering score depending on the clustering parameter is shown. In our case the elbow score is used. This graph is used to select the number of clusters. For elbow method k=the optimal number of clusters is at the point of max score plot curvature. For max interclass variation the optimal number of clusters is at the point of max score plot value. Additionally, user can press the "Export Score Graph" button to save the score values to a .csv file.

5.3.2 Inspection section

The inspection section is used to evaluate the clustering visually. This section contains a drop-down list with widgets: "Metric Distribution", "Reference Grouping Intersection" and "View Spines in 3D".

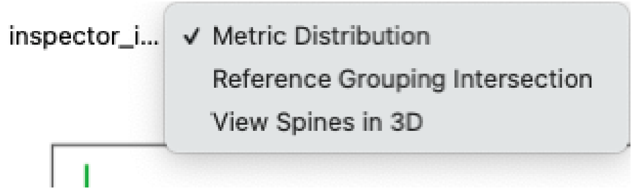

The "Metric Distribution" widget contains graphs of distributions within a cluster for each of the dataset metrics. For chords this is an averaged histogram, for numeric metrics it is a boxplot on values within the cluster.

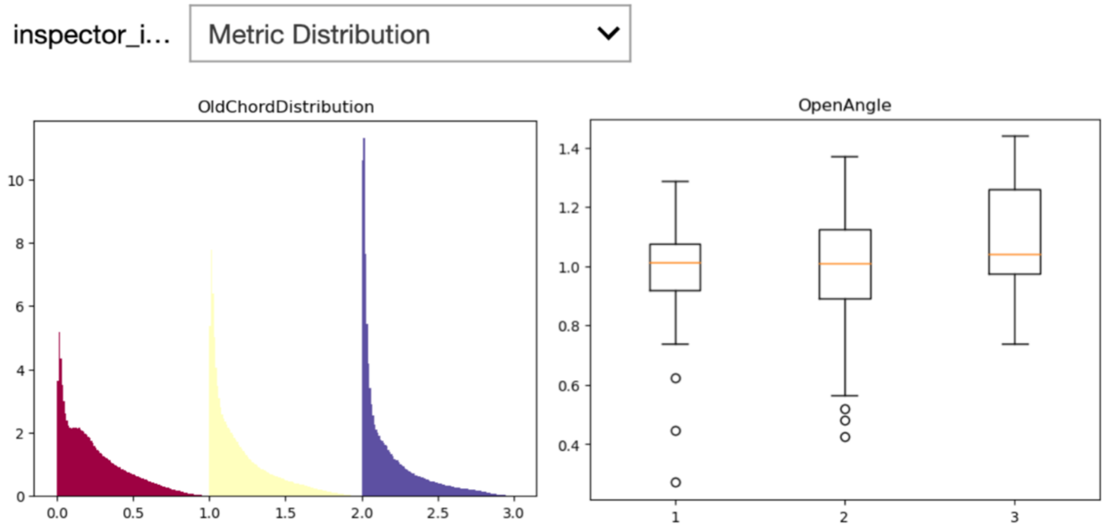

The "Reference Grouping Intersection" widget displays the consistency between two partitions into clusters - the reference one and the obtained one. As the reference partitioning the manual expert classification is used.

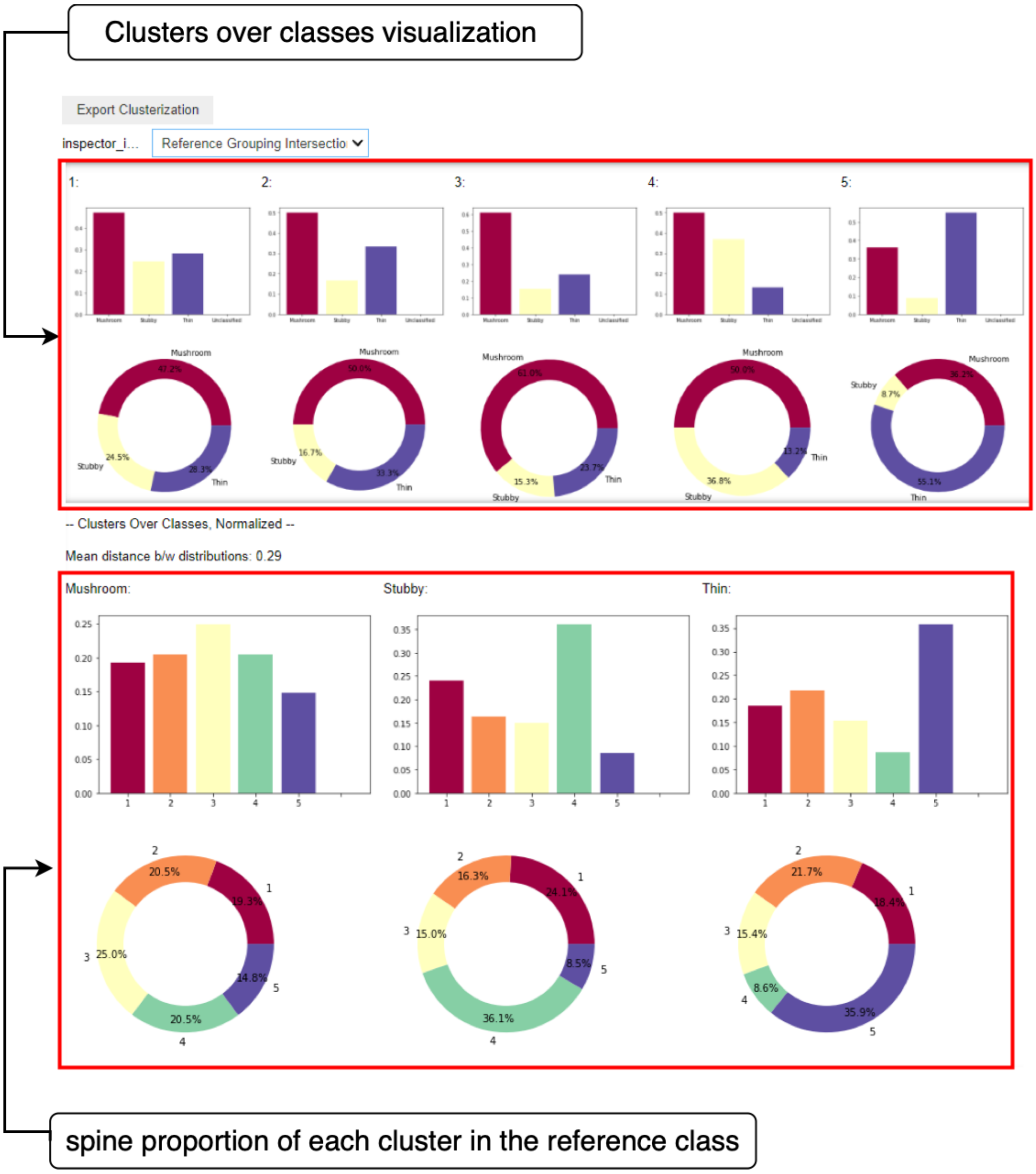

The "View Spines in 3D" widget provides the ability to view spines by cluster. The user selects a cluster he wants to examine from the drop-down menu. A drop-down list of spines belonging to the selected cluster is displayed below.

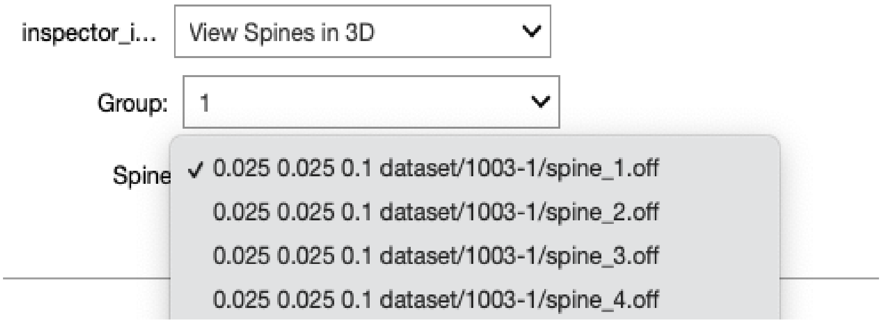

For the selected spine its polygonal grid is drawn, its position on the dendrite grid is highlighted and metric values for this spike are shown.

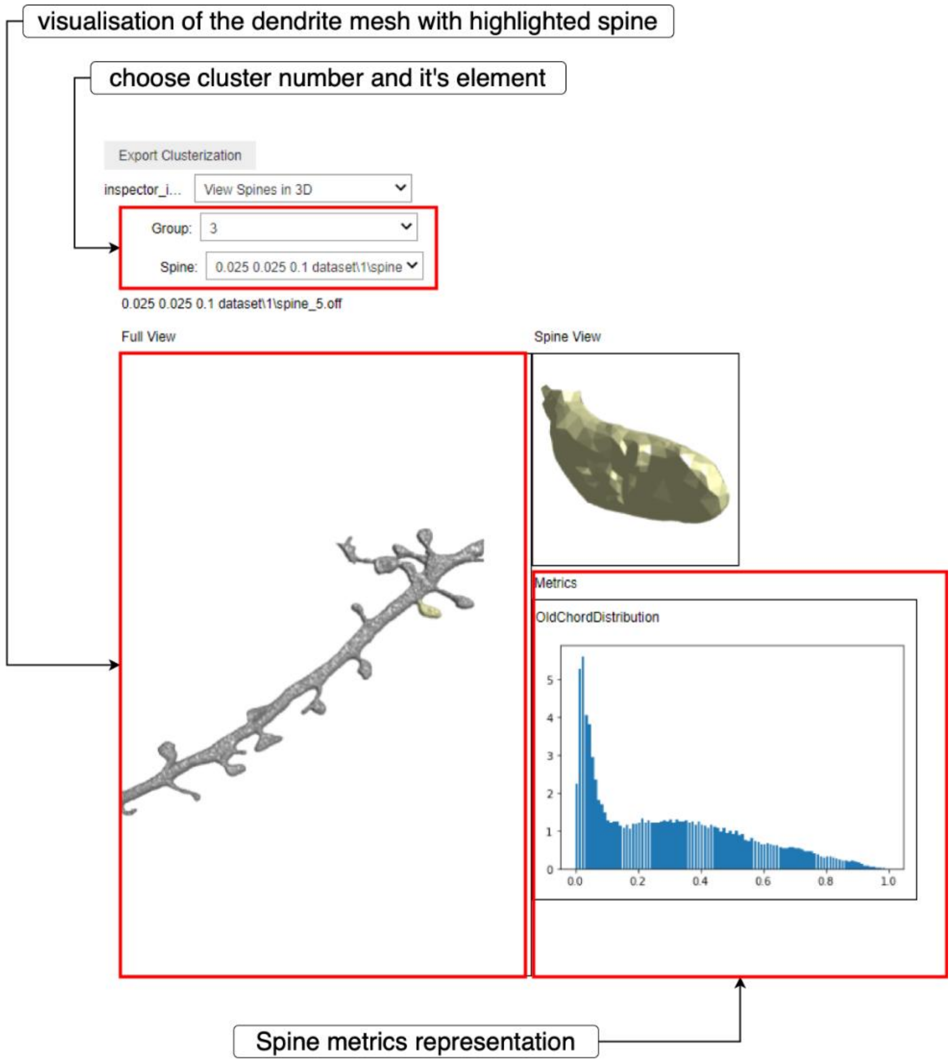

5.3.3 Export section

You can save the resulting clustering by pressing the "export" button. The path where the result will be saved is constructed from the path to folder which is passed as a parameter to the clustering widget and from the clustering parameters – the PCA usage, clustering parameter value and number of clusters.

The path to the output directory is constructed as follows:

```
{save_folder argument passed to clusterizer}/{parameter name: k or eps}={parameter value}_pca={pca argument passed to clusterizer}_{number of clusters}_clusters/.
```

The output directory stores the partitioning of spines into clusters in the clusterization.json file, coordinates of spines after applying the principal component analysis in the pca.csv file, distribution of metrics across clusters in the metric_distributions.csv file and data from comparative analysis of the reference partition with the clustering partition obtained during clustering.

The partitioning is saved to a json file with the following structure: the file has a "groups" section containing sections with names corresponding to the names of clusters or classes and with values as arrays of paths to polygonal meshes of spines contained in that group. An enumeration of all the spines used in grouping is listed in the section "samples" in the form of an array of paths to polygonal meshes of spines. The file also has a section "outliers_label"

which contains the name of a class or cluster, which identifies the unclassified spines considered to be outliers. This name coincides with the name of one of the subsections of the "groups" section. An example of grouping 4 spines into 3 groups "First", "Second" and "Third" is below:

```
{
"groups": {
        "First": [ "spine1.off", "spine3.off"],
        "Second": [ "spine4.off"],
        "Third": [ "spine2.off"],
           },
"samples": ["spine1.off", "spine2.off", "spine3.off", "spine4.off"],
"outliers_label": " Third "
}
```

6 Utilities Notebook

The notebook contains auxiliary methods for working with dendritic spines. Notebook is used to calculate metrics for dataset, review segmentation results, visualize dataset and spine metrics and calculate consensus experts classification.

6.1 Required input and preparations

The user should prepare a dataset with polygonal meshes of spines and dendrites, obtained by executing code from the Dendrite Segmentation Notebook. The path to the prepared dataset must be specified in the first block of the Utilities Notebook code in the dataset_path variable.

```
from notebook_widgets import SpineMeshDataset

# set dataset path and scale applied to spines during visualization
dataset_path = "example_dendrite"
scale = (1, 1, 1)
```

6.2 Metrics Calculation

The "Calculate Metrics" section of the notebook allows to collect and save the desired metrics of a data set. The user can set the parameters for CLDH metrics by changing the values of the variables num_of_chords and num_of_bins in the notebook. The calculated metric values will be stored in the dataset directory and will be required for classification and clustering tasks.

Set CLDH metric parameters

```
from spine_metrics import SpineMetricDataset


# chord method parameters
num_of_chords = 30000
num_of_bins = 100

# calculate metrics
metric_names = ["OldChordDistribution", "OpenAngle", "CVD", "AverageDistance",
                "LengthVolumeRatio", "LengthAreaRatio", "JunctionArea",
                "Length", "Area", "Volume", "ConvexHullVolume", "ConvexHullRatio"]
metric_params = [{"num_of_chords": num_of_chords, "num_of_bins": num_of_bins},
                 {}, {}, {}, {}, {}, {}, {}, {}, {}, {}, {}]
spine_metrics = SpineMetricDataset()
spine_metrics.calculate_metrics(spine_dataset.spine_meshes, metric_names, metric_params)
spine_metrics.save(f"{dataset_path}/metrics.csv")
```

6.3 Dataset Visualization

The notebook contains several sections for data set visualization. It is possible to visualize the dendrite 3d surface (a), dendrite skeleton alone (b) and in surface mesh (c) and the segmentation result (d) for visual result estimation. These views can be obtained by running the code of the "View Dendrite Skeleton" section and the "View Dendrite segmentation" section.

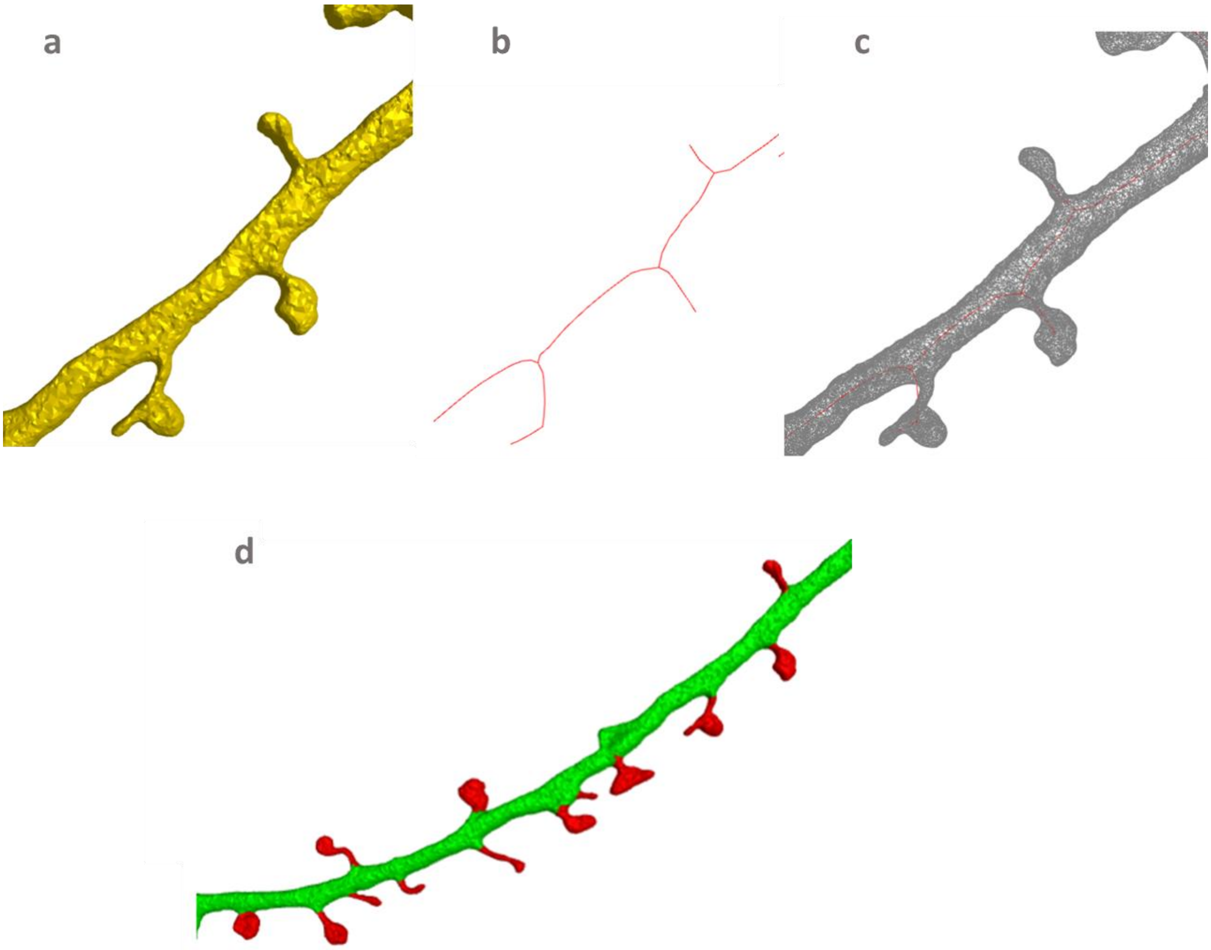

The "View Spines in Dataset" section provides a visualization of the individual spines in the dataset and the view of metrics for them (they have to be computed in 6.2 before that).

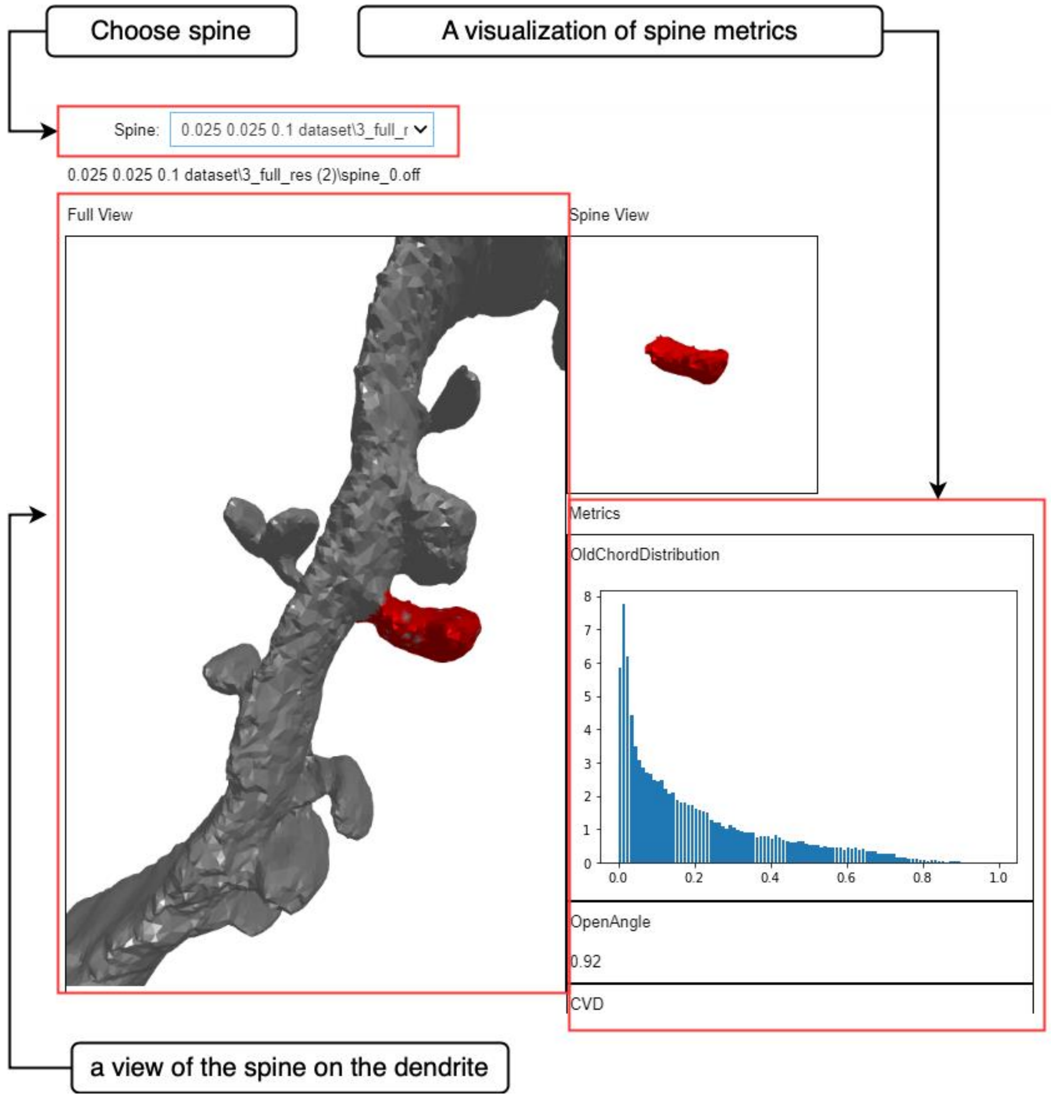

To visualize the set of chords for the spikes, the code of the "View Chords" block should be executed, in which the CLDH descriptor parameters can also be set.

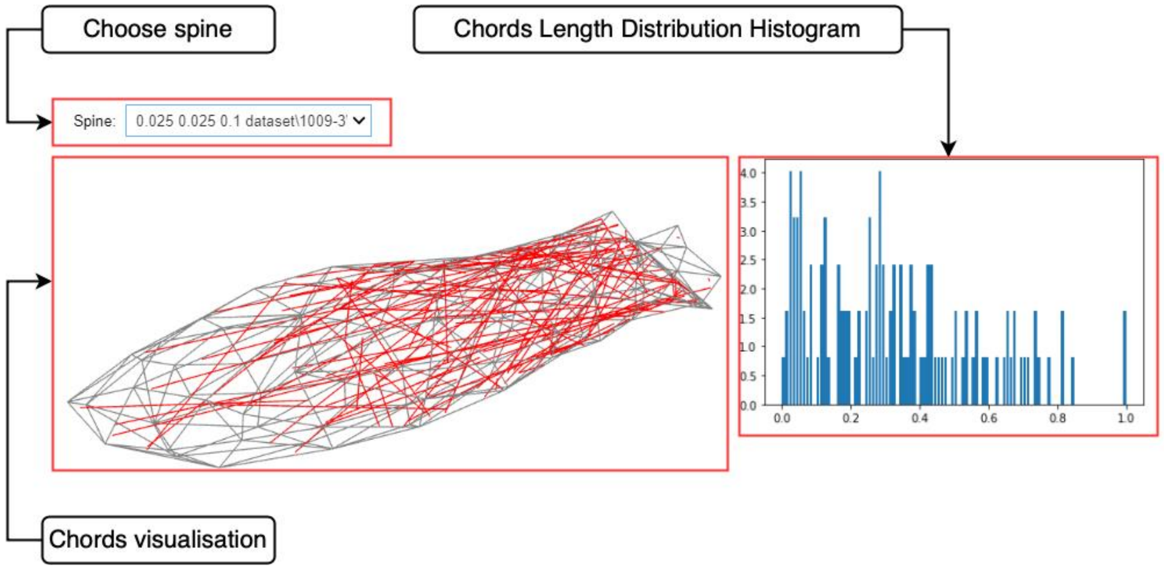

6.4 Creating a consensus classification

To obtain a consensus classification based on the classifications from different experts, the classifications produced by the Manual Classification notebook must be placed in the same subdirectory of the dataset directory, e.g. `PATH_TO_CODE\DATASET_FOLDER\manual_classification\... .json` or specify the full path to the directory with the classifications collected to be processed in the "path" variable.

Specify the path to the classifications to merge

```
from spine_metrics import SpineMetricDataset
from notebook_widgets import create_dir, remove_file
from spine_fitter import SpineGrouping
import pathlib

merged_path = f"{dataset_path}/manual_classification/manual_classification_merged.json"
merged_reduced_path = f"{dataset_path}/manual_classification/manual_classification_merged_reduced.json"

# remove old merged classifications
remove_file(merged_path)
remove_file(merged_reduced_path)

# load manual classifications
path = pathlib.Path(f"{dataset_path}/manual_classification")
classification_paths = [str(classification_path) for classification_path in path.glob("*.json")]
groupings = [SpineGrouping().load(path) for path in classification_paths]
print(f"Merging manual classifications: {classification_paths}\n")

# merge classifications
merged_grouping = SpineGrouping.merge(groupings, outliers_label="Unclassified")
merged_grouping.save(merged_path)
[illegible]
```
